# Enhanced activity of hyperthermostable *Pyrococcus horikoshii* endoglucanase in superbase ionic liquids

**DOI:** 10.1007/s10529-022-03268-5

**Published:** 2022-06-28

**Authors:** Hakim Hebal, Joonas Hämäläinen, Laura Makkonen, Alistair W. T. King, Ilkka Kilpeläinen, Sandip Bankar, Nawel Boucherba, Ossi Turunen

**Affiliations:** 1 Laboratoire de Microbiologie Appliquée (LMA), Faculté des Sciences de La Nature et de La Vie (FSNV), Université de Bejaia, Bejaia, Algeria; 2 Faculty of Exact Sciences and Sciences of Nature and Life, Department of Biology, Mohamed Khider University of Biskra, Biskra, Algeria; 3 St1 Oy, Firdonkatu 2, PL 68, 00521 Helsinki, Finland; 4 Department of Bioproducts and Biosystems, School of Chemical Engineering, Aalto University, 00076 Aalto, Finland; 5 Department of Chemistry, University of Helsinki, 00014 Helsinki, Finland; 6 School of Forest Sciences, University of Eastern Finland, 80101 Joensuu, Finland

**Keywords:** Hyperthermostable endoglucanase, Enzyme kinetics, Enzyme inhibition, Ionic liquids, Biomass engineering

## Abstract

**Objectives:**

Ionic liquids (ILs) that dissolve biomass are harmful to the enzymes that degrade lignocellulose. Enzyme hyperthermostability promotes a tolerance to ILs. Therefore, the limits of hyperthemophilic *Pyrococcus horikoschii* endoglucanase (PhEG) to tolerate 11 superbase ILs were explored.

**Results:**

PhEG was found to be most tolerant to 1-ethyl-3-methylimidazolium acetate ([EMIM]OAc) in soluble 1% carboxymethylcellulose (CMC) and insoluble 1% Avicel substrates. At 35% concentration, this IL caused an increase in enzyme activity (up to 1.5-fold) with CMC. Several ILs were more enzyme inhibiting with insoluble Avicel than with soluble CMC. *K*_m_ increased greatly in the presence ILs, indicating significant competitive inhibition. Increased hydrophobicity of the IL cation or anion was associated with the strongest enzyme inhibition and activation. Surprisingly, PhEG activity was increased 2.0–2.5-fold by several ILs in 4% substrate. Cations exerted the main role in competitive inhibition of the enzyme as revealed by their greater binding energy to the active site.

**Conclusions:**

These results reveal new ways to design a beneficial combination of ILs and enzymes for the hydrolysis of lignocellulose, and the strong potential of PhEG in industrial, high substrate concentrations in aqueous IL solutions.

**Supplementary Information:**

The online version contains supplementary material available at 10.1007/s10529-022-03268-5.

## Introduction

As the main component of biomass, lignocellulose is comprised of cellulose, hemicellulose and lignin (Mckendry 2002). Cellulose is the most abundant polysaccharide on the planet and is, therefore, considered a potentially important source of carbohydrate fibres, and monosaccharides, which can be then used as a substrate for microbial fermentations to produce fuels and value-added chemicals (Chacón et al. 2021; Puligundla et al. 2021). Cellulose is a linear polymer of *β*-1,4 linked-D-glucose units with varying degrees of polymerisation, and is hydrolysed by a consortium of enzymes to glucose: endo-1,4-*β*-glucanase (EC3.2.1.4), cellobiohydrolase (EC 3.2.1.91) and *β*-glucosidase (EC 3.2.1.21). Endo-1,4-*β*-glucanase acts by randomly hydrolysing cellulose chains (Barbosa et al. 2020). However, cellulose is recalcitrant to enzymatic hydrolysis requiring therefore physicochemical pretreatment to decrease its crystallinity and degree of polymerisation, in one hand, and improving of enzyme efficiency, in the other hand (Alvira et al. 2010; Raulo et al. 2021; Contreras et al. 2020).

Ionic liquids (ILs) are salts that exist in liquid form below 100 °C and have the capacity to dissolve lignocellulose for improved hydrolysis by enzymes. They are potentially low polluting and display high thermal and chemical stability, high polarity and very low toxicity (Patel et al. 2014). Dissolved cellulose can be precipitated away from the IL solution using an antisolvent. However, traces of ILs (in the range of 10–15%) may remain in the substrate after precipitation (Engel et al. 2010), and most commercial cellulases are inhibited or deactivated by even low amounts of ILs (Wahlström and Suurnäkki 2015; Nemestóthy et al. 2017; Hebal et al. 2021). Therefore, the discovery or development of new enzymes with greater tolerance towards ILs is a prerequisite for the development of lignocellulose processes that use ILs.

Typically, the highly IL-tolerant endoglucanases and *β*-glucosidases originate from hyperthermophilic microbes (Hebal et al. 2021), which suggests a link between enzyme thermostability and IL tolerance (Ferdjani et al. 2011; Ilmberger et al. 2012). A hyperthermophilic GH5 endoglucanase (PhEG) with the capability of hydrolysing crystalline cellulose from the hyperthermophilic archaon *Pyrococcus horikoshii* has been reported previously (Ando et al. 2002). The extreme thermostability associated with this enzyme (Kang and Ishikawa 2007; Hämäläinen et al. 2016) is probably related to its greater tolerance to ILs. The enzyme was found to retain 90% of its specific activity at 20% 1-ethyl-3-methylimidazolium acetate ([EMIM]OAc) concentration at 80 °C, and 79% of activity was recovered after 15 h of incubation at 15% [EMIM]OAc at 80 °C (Datta et al. 2010). Furthermore, PhEG has been found to be the most IL-tolerant enzyme across a range of studied cellulases in the presence of a 40% concentration of seven cellulose-dissolving or swelling ILs (Rahikainen et al. 2018).

The objective of the current study is to study the effect of diluted aqueous solutions of a wide range of superbase ILs on the functioning of the hyperthermostable PhEG. Definition of a superbase includes *a neutral organic base that is more basic than sodium hydroxide* (Ishikawa 2009). Detailed study of the hyperthermostable enzymes that resist protein denaturation by ILs is expected to better reveal how the enzyme activity itself is affected by ILs. Acetate-propionate ILs show rapid dissolution of cellulose and are potentially distillable (King et al. 2011; Parviainen et al. 2013), while guaiacolate ILs do not dissolve biomass (Hebal et al. 2020). In this study, special attention was paid to enzyme performance on a soluble cellulose substrate (carboxymethyl cellulose, CMC) and an insoluble cellulose substrate (Avicel).

## Materials and methods

### Sources of enzymes and ionic liquids

*Pyrococcus horikoshii* endoglucanase (PhEG) (family GH5) was expressed from *Escherichia coli* and partly purified, as described in Hämäläinen et al. (2016). Ionic liquids (Table 1) were prepared, as described in Parviainen et al. (2013, 2014) and Hebal et al. (2020). [EMIM]OAc was purchased from BASF (Ludwigshafen, Germany, purity ≥ 95%). The superbase-derived ILs were prepared by combining several superbases ([DBNH]^+^, [DBUH]^+^, [mDBN]^+^ and [TMGH]^+^) with organic acids, such as acetate, propionate and guaiacolate (Table 1).

**Table 1:** The tested biomass-dissolving ionic liquids

Ionic liquid		Molecular weight (g/mol)
[DBNH]OAc	1,5-Diazabicyclo[4.3.0]non-5-enium acetate	125.18 + 59.04 = 184.22
[DBNH]CO_2_Et	1,5-Diazabicyclo[4.3.0]non-5-enium propionate	125.18 + 73.07 = 198.25
[DBNH]guaiacolate	1,5-Diazabicyclo[4.3.0]non-5-enium 2-Hydroxy-3-methoxybenzoate	125.18 + 124.13 = 249.31
[DBUH]OAc	1,8-Diazabicyclo[5.4.0]undec-7-enium acetate	153.23 + 59.04 = 212.27
[DBUH]CO_2_Et	1,8-Diazabicyclo[5.4.0]undec-7-enium propionate	153.23 + 73.07 = 226.3
[DBUH]guaiacolate	1,8-Diazabicyclo[5.4.0]undec-7-enium 2-Hydroxy-3-methoxybenzoate	153.23 + 124.13 = 277.36
[EMIM]OAc	1-Ethyl-3-methylimidazolium acetate	111.17 + 59.04 = 170.21
[mDBN]Me_2_PO_4_	Methyl-1,5-diazabicyclo[4.3.0]non-5-enium dimethyl phosphate	139.22 + 126.05 = 265.27
[TMGH]OAc	1,1,3,3-Tetramethylguanidinium acetate	116.18 + 59.04 = 175.22
[TMGH]CO_2_Et	1,1,3,3-Tetramethylguanidinium propionate	116.18 + 73.07 = 189.25
[TMGH]guaiacolate	1,1,3,3-Tetramethylguanidinium 2-Hydroxy-3-methoxybenzoate	116.18 + 124.13 = 240.31

### Enzyme assays

Enzyme activity on soluble cellulose (Na-CMC; Sigma-Aldrich, St. Louis, Montana) and crystalline cellulose (Avicel PH-101; Sigma-Aldrich) were measured with the 3,5-dinitrosalicylic acid (DNS) method (Miller 1959), as described in Hebal et al. (2020). The endoglucanase acted on 1% or 4% (m/v) substrates in 50 mM citrate–phosphate buffer at pH 6 for 30 min at 70 °C, in the absence or presence of 5–35% (v/v) IL solutions. Reciprocal shaking at 200 rpm was used in the enzyme reaction with Avicel and the mixture was centrifuged before the absorbance was measured. The specific activity of the enzymes used in the assays was 14 U/mg with CMC as substrate. One unit (U) of enzyme activity was defined as the amount of enzyme required to liberate 1 μmol of product per min. Since ILs induce an increase in the absorbance, calibration graphs in the presence of ILs were constructed to correct the observed absorbance values (Supplementary Fig. 1).

### Kinetic experiments

Kinetic parameters were determined in standard assay conditions at 70 °C (pH 6) with and without 5% (v/v) IL. Initial velocities were determined using 4, 6, 10 and 20 mg/ml CMC at different incubation times (10, 20, 30 and 40 min). Initial velocity was determined from the slope of the graph (Supplementary Fig. 2 and Supplementary Fig. 3). All experiments were performed three times with triplicates. Kinetic values were calculated by the hyperbolic regression analysis function (without weighting) in the Hyper 32 program.

### Molecular docking

The IL cations and anions were docked by SwissDock (Grosdidier et al. 2011) to the active site of PhEG (PDB code 2ZUM; Kim and Ishikawa 2009), as described in Hebal et al. (2020).

## Results and discussion

The use of ILs in combination with enzymes profoundly affects the kinetic behaviour and stability of enzymes. Therefore, in this study, the kinetic behaviour of an hyperthermostable endoglucanase was studied in aqueous IL solutions. The goal was to obtain a better picture of the competitive inhibition caused by ILs with regard to the variation of IL cations and the anions, and the nature of the substrate. Although several glycoside hydrolase enzymes (including PhEG) have been studied earlier, our study revealed new behaviour associated with a hyperthermostable endoglucanase in a range of ILs.

### Activity of PhEG in 5–35% ionic liquids

**Fig. 1:**
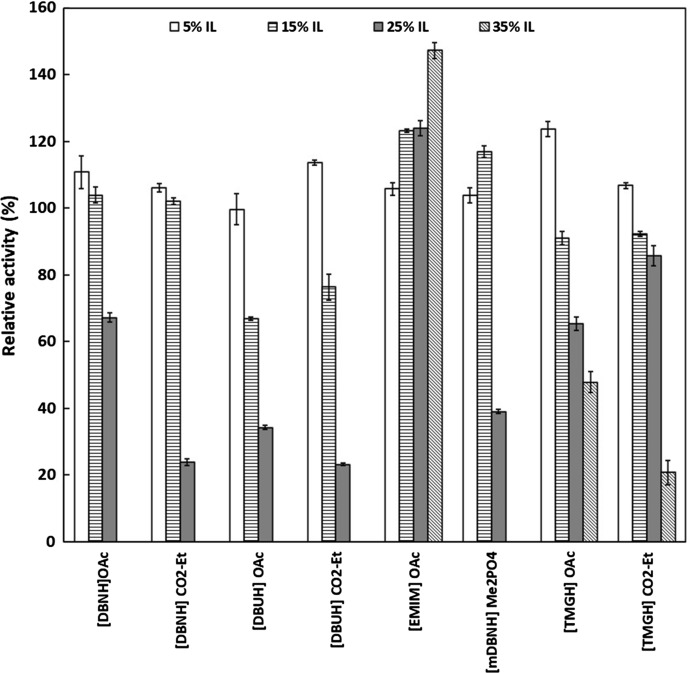
Activity of *Pyrococcus horikoschii* endoglucanase (PhEG) in 5–35% ionic liquids with 1% carboxymethylcellulose (CMC) as substrate. Activity was measured at 70 °C and pH 6. 100% activity denotes the activity without IL

**Fig. 2:**
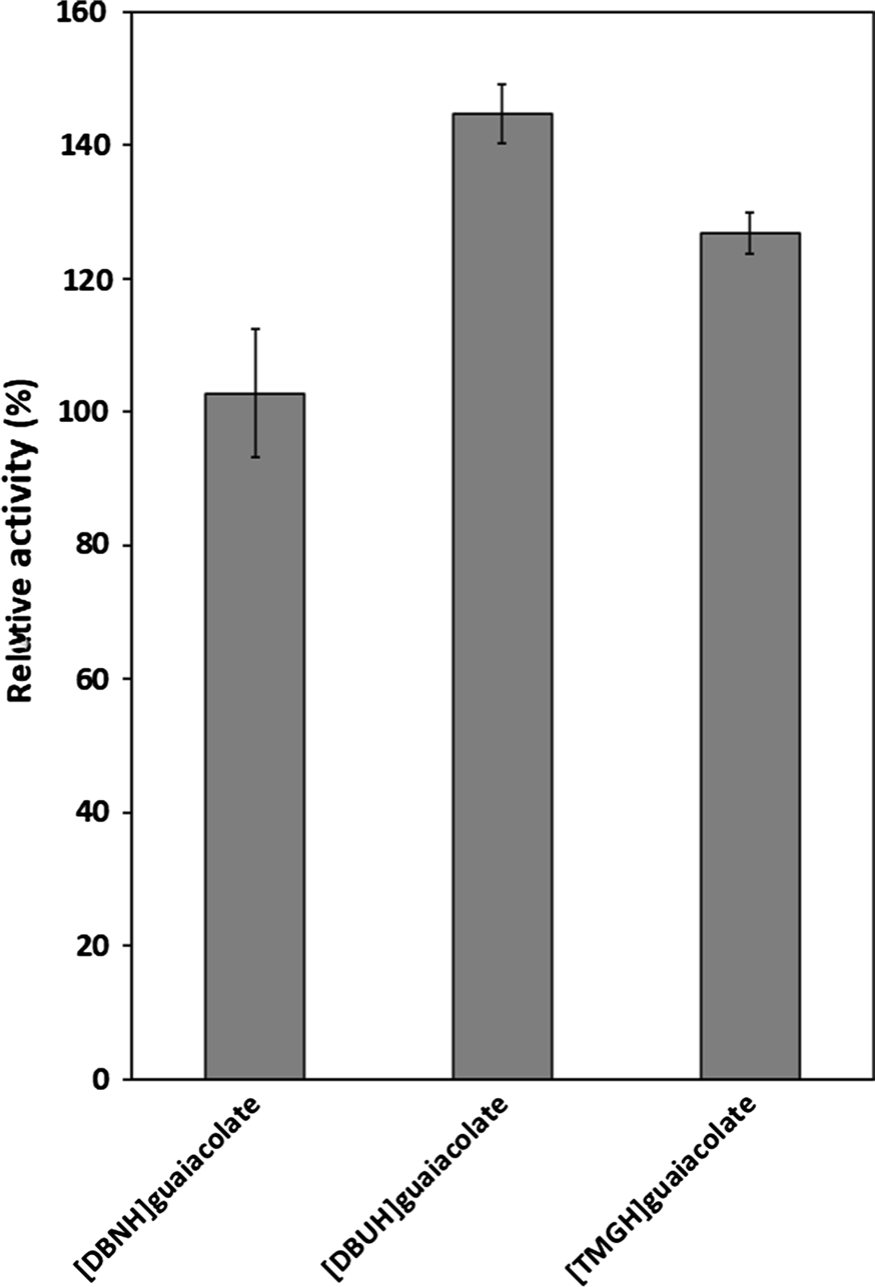
Activity of *Pyrococcus horikoschii* endoglucanase (PhEG) in 5% guaiacolate ionic liquids with 1% carboxymethylcellulose (CMC) as substrate. Activity was measured at 70 °C and pH 6. 100% activity denotes the activity without IL

The activity of PhEG was studied in 5–35% acetate-propionate ILs (Fig. 1), and in 5% guaiacolate-based ILs (Fig. 2), with 1% CMC as a substrate. In acetate-propionate ILs (Fig. 1), [EMIM]OAc was the only IL that favoured PhEG activity at higher IL concentrations. An increasing concentration of [EMIM]OAc up to 35% increased enzyme activity. Other ILs at 25–35% concentrations greatly decreased enzyme activity. Only [EMIM]OAc, [TMGH]OAc and [TMGH]CO_2_Et showed activity at 35% concentration, although the latter two reduced the activity. At 15% concentration, [mDBN]Me_2_PO_4_ also showed clear elevation of PhEG activity. Altogether, [DBNH]OAc, [DBNH]CO_2_Et and [mDBN]Me_2_PO_4_ were well tolerated by PhEG at 15% IL concentration, but activity was rapidly lost at 25% concentration. No consistent trend of inhibition was observed at 25% IL concentration when the cation or the anion were varied (Fig. 1). These superbase ILs exerted a clear inhibition on the hyperthermostable *Thermopolyspora flexuosa* GH10 xylanase TfXYN10A at this concentration (Hebal et al., 2020). At 35% ILs, PhEG seemed to be most inhibited by hydrophobic cations, i.e. ([DBNH]^+^, [DBUH]^+^ and [mDBN]^+^), and was less inhibited or even stimulated by the less hydrophobic cations ([EMIM]^+^, [TMGH]^+^). A similar finding was observed when TfXYN10A was assayed with superbase ILs (Hebal et al. 2020). However, in general, TfXYN10A seems to be more inhibited by superbase ILs than PhEG at the same range of IL concentrations. In addition, PhEG was most inhibited by the [DBUH]^+^ cation, whereas TfXYN10A was more inhibited by [DBNH]^+^ than [DBUH]^+^ cation. Wahlstrom et al. (2013) studied the hydrolysis of microcrystalline cellulose (MCC) by *Trichoderma reesei* endoglucanase Cel5A in the presence of 20% (w/w) superbase ILs. In line with our results, the authors found that the greatest MCC hydrolysis yield was obtained with [EMIM]AOc, followed by [TMGH]AOc, and that the activity was strongly inhibited by [DBNH]OAc and [DBNH]CO_2_Et.

When ILs were used at 5% concentration, a slight increase in enzyme activity was observed in most acetate-propionate ILs but especially in [TMGH]OAc, [DBUH]CO_2_Et and [DBNH]OAc (Fig. 1). Of these, [TMGH]OAc induced the greatest increase in activity. Enzyme activity was enhanced with guaiacolate IL when the cations were [DBUH]^+^ and [TMGH]^+^ but not with [DBNH]^+^ (Fig. 2). In contrast, 5% guaiacolate ILs inhibited hyperthermostable GH10 and GH11 xylanases (Hebal et al. 2020). The unconjugated cations displayed the following degrees of declining basicity: [mDBN]^+^  > [EMIM]^+^  > [DBUH]^+^  > [DBNH]^+^  > [TMGH]^+^ (Parviainen et al. 2013). The more basic cations, [mDBN]^+^ and [EMIM]^+^ are probably more prone to interact with water molecules than with protein and, therefore, exert a less activating effect on the enzyme. No consistent trend in enzyme activation was observed for the anions.

Our results for PhEG activity in [EMIM]OAc are, to some degree, different from those reported by Datta et al. (2010), since [EMIM]OAc was observed to activate the enzyme in our study. However, experimental conditions were different in these two studies. In Datta et al. (2010), a temperature of 80 °C was used in the enzyme reaction compared to 70 °C in our study. Moreover, the use of bovine serum albumin (BSA) as a stabiliser and pH adjustment after IL addition was not reported by Datta et al. (2010) (initial pH was 6.4 in Datta et al. (2010); adjusted pH was 6 in our study). Other studies have shown that ILs can decrease the temperature optimum of enzyme activity (Yu et al. 2016; Anbarasan et al. 2017) and modify the pH of the reaction mixture, while stabilizing agents may also increase the tolerance of enzymes to ILs (Zhao 2010). It is probable that our experimental conditions better supported the functioning of the enzymes in ILs. These differences in the results suggest that reaction condition engineering is important in improving enzyme tolerance to ILs. The influence of reaction conditions on enzyme tolerance to ILs was also seen with TfXYN10A, since [EMIM]OAc stabilised the enzyme at 80 °C but not at 60–70 °C (Anbarasan et al. 2017).

In particular, the stimulation of PhEG at 35% [EMIM]OAc, could contribute to the simplification of the overall IL-pretreatment-hydrolysis process by allowing the enzymatic saccharification of cellulose by eliminating the need to recover the regenerated cellulose (Kamiya et al. 2008). In this strategy, after the IL pretreatment of lignocellulose, a buffer solution is added to decrease the IL concentration to < 50%, followed by addition of the IL tolerant enzymes. This would overcome the challenges caused by viscosity of the solution and the excessive washing required to remove the ILs and should, therefore, reduce the process costs. However, the one pot process with ILs seems not to be possible with existing commercial cellulases, since it has been shown that [EMIM]OAc concentrations > 10% compromise the performance of these enzymes (Auxenfans et al. 2017; Husson et al. 2018).

PhEG was slightly activated in the presence of 40% [TMGH]n-PrCOO (Rahikainen et al. 2018), and enzyme activation by ILs has also been observed in other hyperthermophilic endoglucanases (Hebal et al. 2021). This phenomenon has been proposed to reflect the need for salt to achieve maximal enzyme activity (Ajloo et al. 2013; Gladden et al. 2014). However, PhEG activity was not stimulated with NaCl or KCl (0.5–4 M) in this study (Supplementary Fig. 4). On the contrary, the activity of a halophilic endoglucanase that was enhanced by NaCl was not stimulated by ILs (Ben Hmad et al. 2017). This suggests a different mechanism of enzyme activation by ILs and that other types of IL-protein interactions are important, in addition to electrostatic effects. In this respect, a major property of ILs is the hydrophobic portion of the cations.

Protein denaturation has been shown to be absent or minimal for several hyperthermostable glycoside hydrolases in dilute IL solutions (Hebal et al. 2021). The unfolding temperature of PhEG only decreased by 10.5 °C in 20% [EMIM]OAc, from 102.3 to 91.8 °C (Datta et al. 2010). Only minimal structural changes were observed in this enzyme when simulated in the presence of 15% and 50% [EMIM]OAc at 80 °C (Jaeger et al. 2015). These results suggest that the enzyme is not very prone to unfolding at the assay temperature of 70 °C used in our study, and it is likely that the ILs affect the enzyme by inhibiting activity rather than by unfolding the protein. In several studies, loss of hyperthermostable enzyme activity in dilute IL solutions was found to be caused by competitive inhibition of IL molecules, especially the cation, during the binding of the substrate to the active site (Hebal et al. 2021).

### Activity of PhEG in 4% substrate

**Fig. 3:**
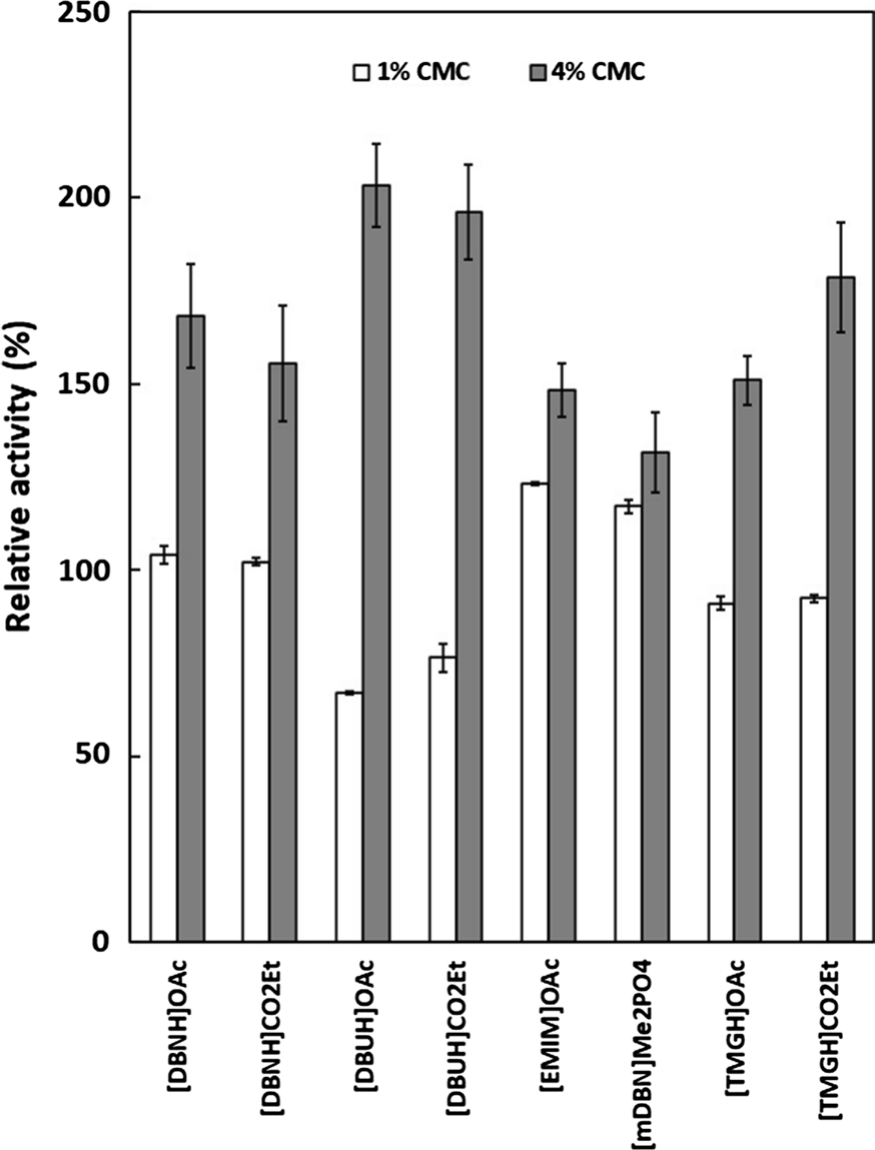
Activity of *Pyrococcus horikoschii* endoglucanase (PhEG) in 1% and 4% substrates in the presence of 15% ionic liquids. Activity was measured at 70 °C and pH 6. 100% activity denotes the activity in each substrate concentration without IL

**Fig. 4:**
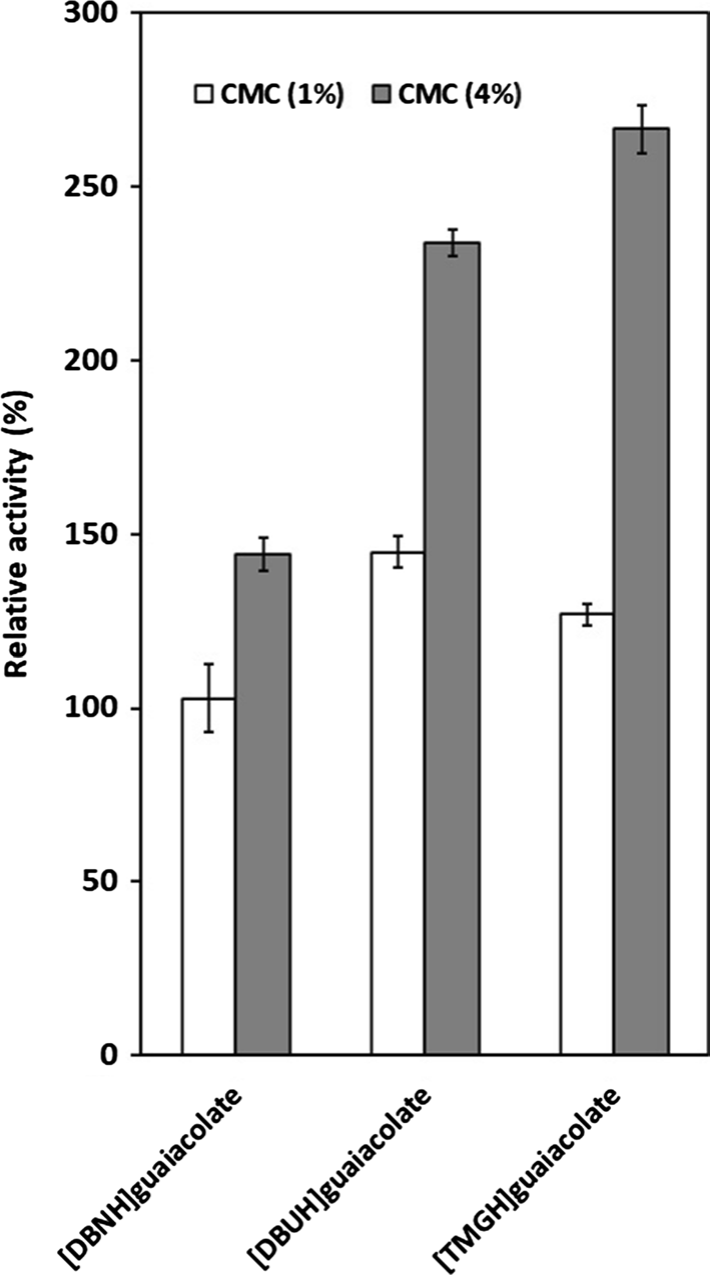
Activity of *Pyrococcus horikoschii* endoglucanase (PhEG) in 1% and 4% substrates in the presence of 5% guaiacolate ionic liquids. Activity was measured at 70 °C and pH 6. 100% activity denotes the activity in each substrate concentration without IL

To clarify the role of competitive inhibition, the effect of ILs on PhEG activity was compared in 1% and 4% substrate concentrations in the presence of 15% acetate-propionate ILs, [mDBN]Me_2_PO_4_ (Fig. 3) and 5% guaiacolate ILs (Fig. 4). In competitive inhibition, the greater substrate concentration counteracts inhibition by outcompeting the inhibitor. With PhEG, increasing the substrate concentration resulted not only in full recovery of activity but also in the activation of PhEG with all acetate-propionate ILs and [mDBN]Me_2_PO_4_ (Fig. 3). With guaiacolate ILs, an increase in substrate concentration further stimulated enzyme activity, with [TMGH]guaiacolate exerting the most stimulating effect (Fig. 4). The elevated xylan concentration also counteracted the competitive inhibition of TfXYN10A by superbase ILs (Hebal et al. 2020) and of TasXyn10A by 25% [EMIM]OAc (Chawachart et al. 2014). Increased activity in the presence of elevated substrate concentrations suggests that competitive inhibition was counteracted by the substrate. However, the noteworthy increase observed in PhEG activity in 4% substrate with ILs may even indicate that IL molecules in high substrate concentrations stimulate the reaction interaction between the substrate and the enzyme. These results reveal that the IL molecules may both activate and inhibit the enzymes, and that the balance between these two opposing behaviours depends at least partly on the conditions.

Unlike PhEG under the same conditions, TfXYN10A was not activated or was only slightly activated by superbase ILs in 4% substrate (Hebal et al. 2020). This difference suggests that superbase ILs exert a differing effect on PhEG compared to TfXYN10A. No difference between acetate and propionate was observed when activating PhEG in 4% substrate when they were conjugated to more hydrophobic cations ([DBNH]^+^ and [DBUH]^+^). Propionate and guaiacolate showed greater activation than acetate when they were conjugated to the less hydrophobic cation [TMGH]^+^ (Figs. 3 and 4). These results suggest that increased hydrophobicity of the IL ions is among the main factors that contribute to the activation of PhEG, and that activation by the anion depends on the degree of hydrophobicity of the cation. With TfXYN10A, the acetate anion caused minor activation in the 4% substrate, while the propionate and the guaiacolate ILs did not cause any significant activation (Hebal et al. 2020). With [DBUH]OAc and [DBUH]CO_2_Et, the relative PhEG activity was doubled with 4% CMC when compared to the level without ILs (Fig. 3). Similarly, the activity more than doubled with [TMGH]guaiacolate and [DBUH]guaiacolate (Fig. 4). With such enhanced activity, the use of these types of ILs would be advantageous in industrial, high substrate conditions.

### Comparison of PhEG activity in CMC and avicel

**Fig. 5:**
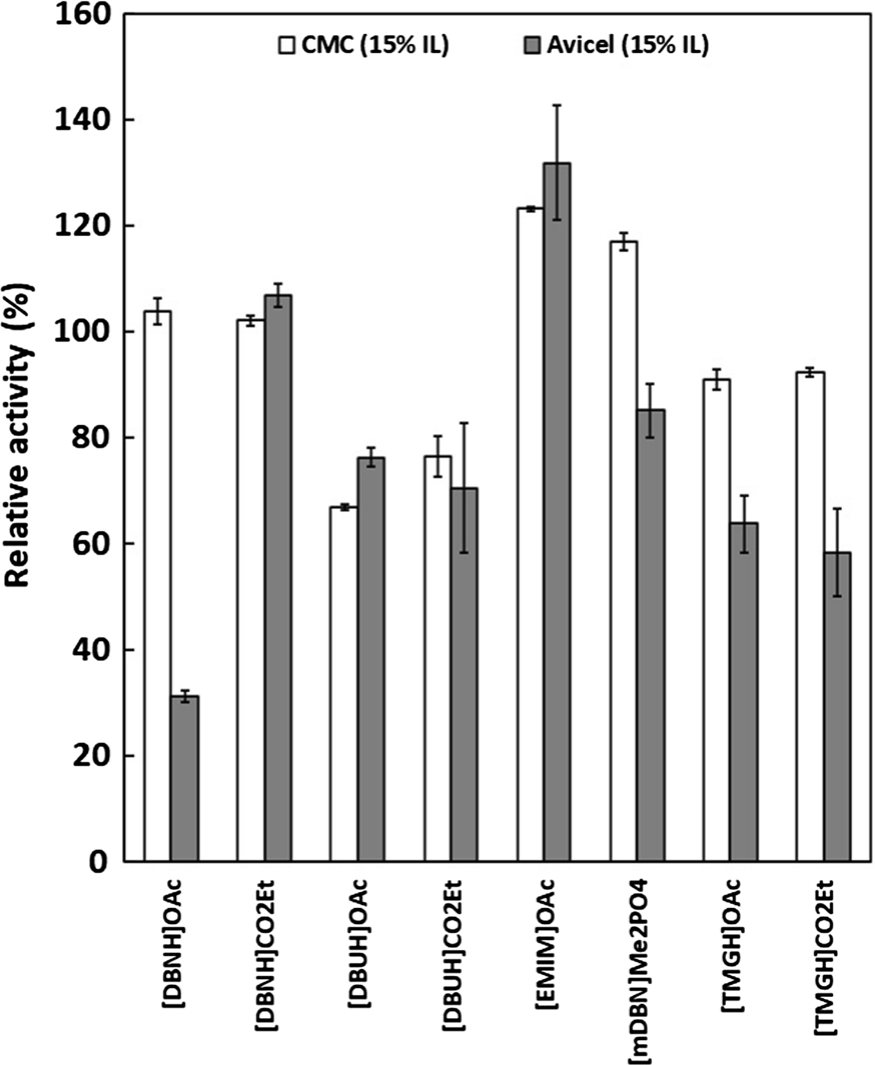
Comparison between activities of *Pyrococcus horikoschii* endoglucanase (PhEG) on 1% carboxymethylcellulose (CMC) and 1% Avicel in the presence of 15% ionic liquids. Activity was measured at 70 °C and pH 6. 100% activity denotes the activity without IL

**Fig. 6:**
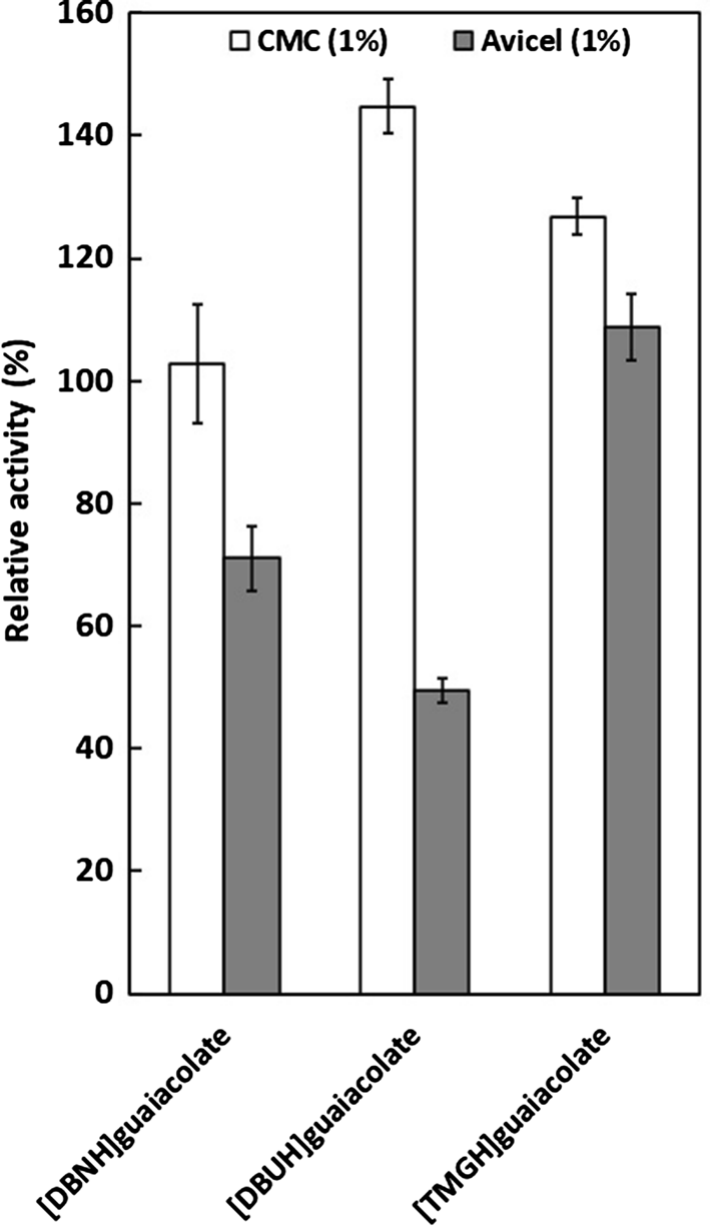
Comparison between activities of *Pyrococcus horikoschii* endoglucanase (PhEG) on 1% carboxymethylcellulose (CMC) and 1% Avicel in the presence of 5% guaiacolate ionic liquids. Activity was measured at 70 °C and pH 6. 100% activity denotes the activity without IL

Since PhEG can hydrolyse both soluble cellulose (CMC) and insoluble microcrystalline cellulose (Avicel), we tested how ILs affect enzyme activity with these substrates. Comparison of the two substrates revealed two distinct activity patterns. In [DBNH]CO_2_Et, [DBUH]OAc, [DBUH]CO_2_Et and [EMIM]OAc, enzyme activity was at the same level for both substrates (Fig. 5). These ILs have a greater cellulose solubilising capability compared to the remaining ILs (Parviainen et al. 2013, 2014), which suggests that at least partial Avicel solubilisation or swelling could have occurred, leading to similar relative enzyme activity as observed with soluble CMC. Rahikainen et al. (2018) found that the most endoglucanase-compatible ILs had the greatest potential for pulp fibre swelling. Although [DBNH]OAc has an enhanced cellulose-dissolving capability, only 30% activity was observed on Avicel. This IL formed a viscous solution with Avicel, which could be the reason for the low activity. This observation indicates that some modification in the state of Avicel occurs in these ILs. For the ILs that are less dissolving, i.e. ([mDBN]Me_2_PO_4_, [TMGH]OAc and [TMGH]CO_2_Et), or those that do not dissolve cellulose (i.e. guaiacolate ILs) (Parviainen et al. 2013, 2014; Hebal et al. 2020), the activity on Avicel was lower than the activity on CMC (Figs. 5 and 6). These results directly show the influence of substrate solubility on enzyme tolerance to ILs. Chawachart et al. (2014) reported that the xylanase TasXyn10A was more strongly inhibited with [EMIM]OAc when short molecule pNP-xylose was used as a substrate instead of the longer xylan chains. Work by Hu et al. (2016) showed that inhibition of cellulase by ILs was substrate-dependant. However, the studied cellulase was a mixture of endoglucanases, exoglucanases and *β*-glucosidases, and the IL may differentially affect each enzyme specific for each substrate.

**Fig. 7:**
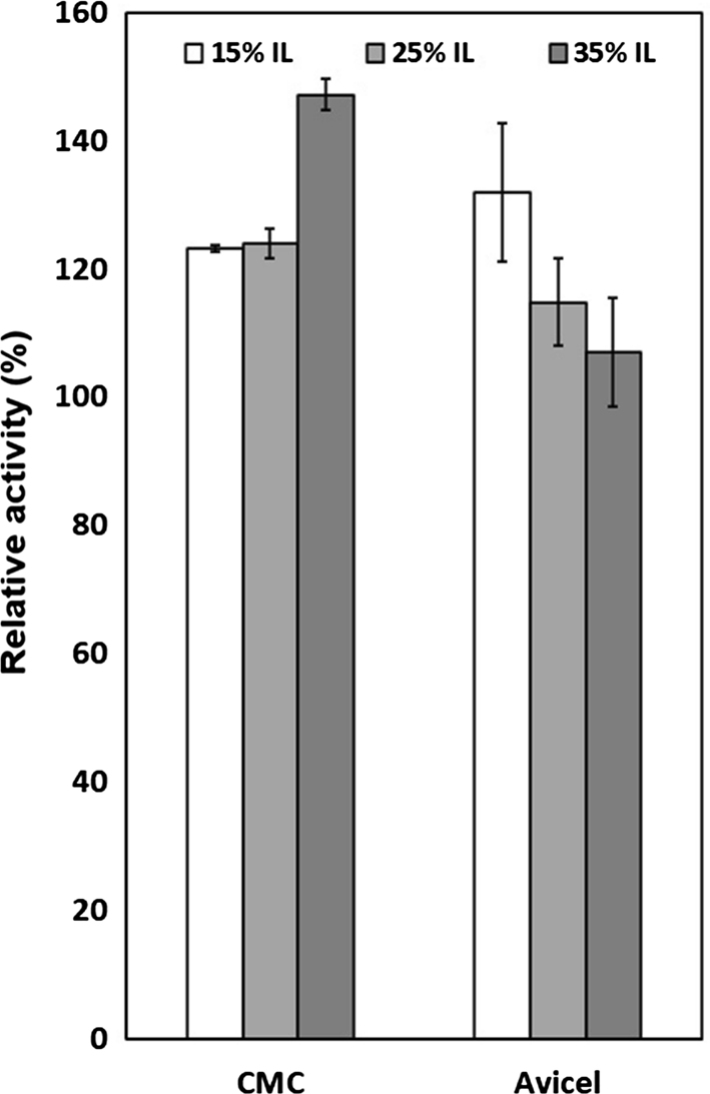
Comparison between activities of *Pyrococcus horikoschii* endoglucanase (PhEG) on 1% carboxymethylcellulose (CMC) and 1% Avicel in the presence of 15–35% [EMIM]OAc. Activity was measured at 70 °C and pH 6. 100% activity denotes the activity without IL

In this study, [EMIM]OAc permitted full activity (even slightly enhanced activity) of PhEG in both substrates at 15% concentration at 70 °C (Fig. 5). In contrast, Datta et al. (2010) observed a 10% decrease in the sugar yield when PhEG hydrolysed pretreated Avicel in the presence of 15% [EMIM]OAc at 80 °C. This difference may be due to the differences in the reaction conditions, as discussed above. In our study, a clear decrease in enzyme activity was observed with Avicel but not with CMC when [EMIM]OAc was increased to 35% concentration (Fig. 7). This decrease in activity suggests that the extent of Avicel solubilisation at 35% concentration was not sufficient to completely overcome the greater competitive inhibition at the more elevated IL concentration.

The tolerance of PhEG to 35% [EMIM]OAc in hydrolysing crystalline cellulose may have practical importance. It has been shown that relatively low [EMIM]OAc concentrations (25–50% w/v) in water may be effective in pretreating biomass (Fu and Mazza 2011). Therefore, it could be possible to perform with IL tolerant enzymes a simple one pot reaction procedure combining simultaneous aqueous IL and enzyme treatments.

### Effect of ionic liquids on the kinetic parameters of PhEG

To further clarify the mechanism by which IL molecules affect enzyme activity, the Michaelis–Menten kinetic parameters *K*_m_ (Michaelis constant) and *V*_max_ (maximum reaction velocity) values of the enzymatic reaction were determined in the presence of 5% ILs (Table 2). *V*_max_ was measured as a relative value regarding the value without ILs, which gives a comparative result for the effect of ILs.

**Table 2:** Effect of 5% ionic liquids on *Pyrococcus horikoshii* endoglucanase (PhEG) kinetic parameters. Activity was measured at 70 °C and pH 6

Ionic liquid	Relative *V*_max_	*K*_m_ (mg/ml)	Relative *V*_max_/*K*_m_
None	1.000 ± 0,002	1.007 ± 0.306	1.064 ± 0.357
[DBNH]OAc	1.495 ± 0.098	3.273 ± 1.136	0.484 ± 0.119
[DBNH]CO_2_Et	1.72 ± 0.085	5.580 ± 0.225	0.308 ± 0.003
[DBNH]guaiacolate	1.418 ± 0,175	4.233 ± 0,92	0.34 ± 0,031
[DBUH]OAc	2.498 ± 0.152	13.466 ± 2.3	0.187 ± 0.02
[DBUH]CO_2_Et	2.697 ± 0.166	7.698 ± 0.768	0.282 ± 0.015
[DBUH]guaiacolate	2.464 ± 0,123	8.474 ± 0,125	0.3 ± 0,016
[EMIM]OAc	1.200 ± 0.104	1.668 ± 0.215	0.724 ± 0.053
[mDBN]Me_2_PO_4_	1.551 ± 0.069	1.955 ± 0.405	0.811 ± 0.13
[TMGH]OAc	1.879 ± 0.104	4.570 ± 0.269	0.413 ± 0.036
[TMGH]CO_2_Et	1.647 ± 0.069	3.760 ± 1.598	0.488 ± 0.177
[TMGH]guaiacolate	3.071 ± 0.342	11.92 ± 0.444	0.258 ± 0.016

In this study, all ILs in 5% concentration increased *K*_m_, which is compatible with the premise that ILs cause competitive inhibition by binding transiently to the active site (Hebal et al. 2021). Among the cations, [DBUH]^+^ conjugated to all anions and [TMGH]^+^ conjugated to guaiacolate, caused the greatest increase in *K*_m_ (Table 2). Moreover, *V*_max_ also increased, but the increase was lower than that observed for *K*_m_, which indicates that ILs had a greater inhibiting than activating effect on the enzyme in these conditions.

There was an approximate linear correlation between the increase in *V*_max_ in the presence of ILs and the increase in enzyme activity with 4% substrate in the presence of ILs (Supplementary Fig. 5). This suggests that *V*_max_ reflects the ability of the enzyme to be stimulated when competitive inhibition is relieved. Similarly, enzyme activity in 25% ILs correlates with the relative catalytic efficiency (relative *V*_max_/*K*_M_) in the presence of ILs (Supplementary Fig. 6), which indicates that the theoretical *K*_m_ and *V*_max_ values in the presence of ILs successfully capture the effects of ILs on enzyme activity. An increase in *K*_m_ tends to lead to an increase in *V*_max_, which was clearly seen in the effect of ILs on PhEG activity (Supplementary Fig. 7).

The increase in both *K*_m_ and *V*_max_ values or *k*_cat_ (catalytic rate constant) has also been observed for a hyperthermostable *β*-glucosidase (Kudou et al. 2014) and other enzymes (Daneshjoo et al. 2011; da Silva and de Castro 2018) in ILs. The increase in *V*_max_ partly mitigates the effect of competitive inhibition when weaker interaction with the enzyme may improve the product release. In addition, if the ILs reshape the active site interactions, this could affect the activity levels, even positively, in the presence of ILs. Yu et al. (2013) concluded that the increase in both *V*_max_ and *K*_m_ values for laccase in aqueous solutions of tetramethylammonium trifluoromethanesulfonate ([TMA]TfO) was associated with a more compact protein structure (resulting from alteration of enzyme surroundings by IL). They suggested that this compact structure hinder the approaching of the substrate to the active site, but also enhances the catalytic rate. A simulation study of GH11 xylanase in an aqueous [EMIM]OAc solution suggested that the strong binding of cations in the enzyme active site may potentially reduce or enhance activity by inducing new geometries or electronic structures that affect the transition states (Jaeger and Pfaendtner 2013). Another simulation study of hyperthrmostable xylanase TmXYN10B in an aqueous [EMIM]OAc solution revealed conformational changes to the active site induced by the [EMIM]^+^ cations (Manna and Ghosh 2021). It is possible that the strong increase in enzyme activity by ILs in elevated substrate concentrations is mediated both by IL interactions with the substrate and the enzyme.

In contrast to PhEG, which experienced even a strong increase in *V*_max_ by 5% ILs, the *V*_max_ value associated with TfXYN10A remained the same, increased only slightly with two ILs or decreased at 5% superbase ILs (Hebal et al., 2020). However, an increase in *V*_max_ was seen for TfXYN10A at elevated concentrations of [EMIM]OAc and [DBNH]OAc (15 and 35%) (Anbarasan et al. 2017). In addition, the *V*_max_ values of other hyperthemostable xylanases, which show less tolerance to [EMIM]OAc (in contrast to PhEG), were not affected or decreased in the presence of this IL (Li et al. 2013; Chawachart et al. 2014; Yu et al. 2016). These results indicate that activation of PhEG, as reflected by the increase in *V*_max_, contributes to a general greater tolerance to superbase ILs of this enzyme. Moreover, PhEG has been found to be partly tolerant to 40% concentration of another set of ILs, i.e. ([DMIM]DMP, [BMIM]DBP, [Chol]AcO, [BMIM]DMP, [TMGH]n-PrCOO, and [EMIM]DMP), and retained full activity (107%) in [TMGH]n-PrCOO (Rahikainen et al. 2018). However, the effects of insoluble Avicel and elevated substrate concentration were not studied by Rahikainen et al. (2018).

The [DBUH]^+^ cation systematically triggered the greatest increase in kinetic parameters with all anions, which indicates the strong effect of this cation on enzyme functioning. In line with this finding, almost no differences were observed in the kinetic parameters between the different anions when conjugated to [DBUH]^+^. This indicates that the studied anions, in general, have a lesser effect on enzyme activity than cations. Anions showed the strongest effect when the larger guaiacolate anion was conjugated to [TMGH]^+^, which is also an indication of the cooperative effect of these two ions in influencing PhEG activity.

[DBUH]^+^ differs from the other cations by its greater hydrophobicity (the amount of hydrophobic C and H atoms: [DBUH]^+^ 25; [mDBN]^+^ 23; [DBNH]^+^ 19; [EMIM]^+^ 17 and [TMGH]^+^ 17), which suggests a role for hydrophobic interactions in the IL interaction with the active site and in activation of the enzyme. Furthermore, PhEG showed lowered activity in the presence of 15% [Chol]OAc (Rahikanen et al. 2018). This IL has lower hydrophobicity compared to the acetate-based ILs used in our study. Differences between [HMIM]^+^ and [BMIM]^+^ ILs in concentrations < 40% in the activation of lipase have been attributed to the difference in the length of the alkyl chain (hydrophobicity) attached to the cation (Daneshjoo et al. 2011). [HMIM]Cl has been reported to increase *K*_m_ and *V*_max_ values more than [BMIM]Cl in IL concentrations < 25%, and enzyme activation has been suggested to result from structural changes induced by ILs in the protein (Daneshjoo et al. 2011).

In a simulation study by Jaeger et al. (2015), PhEG exhibited greater stability at 80 °C in 50% [EMIM]OAc concentration compared to stability in pure water, and the presence of IL resulted in the formation of non-native salt bridges in PhEG. Dilute solutions of [EMIM]OAc also induced stabilisation of hyperthermostable *β*-glucosidase and xylanases (Kudou et al. 2014; Yu et al. 2016; Anbarasan et al. 2017). The stabilisation of a three α-helix bundle in [BMIM]Cl was found to be due to electrostatic and hydrophobic interactions of the [BMIM]^+^ cation with residues on the protein surface, and such interactions could remove the surrounding water molecules, reduce the hydrogen bonding from water to protein, and thus stabilise the backbone hydrogen bonds (Shao, 2013). Although ILs seem to unfold the proteins (Zhao 2016; Smiatek 2017), these results indicate that the effect of ILs on the activity of enzymes may also include stabilisation of the enzyme structure by interactions at the active site and other parts of the protein. When the enzyme is hyperthermostable, the stabilising effect of ILs could become dominating.

### Binding of ionic liquid molecules to the active site

**Fig. 8:**
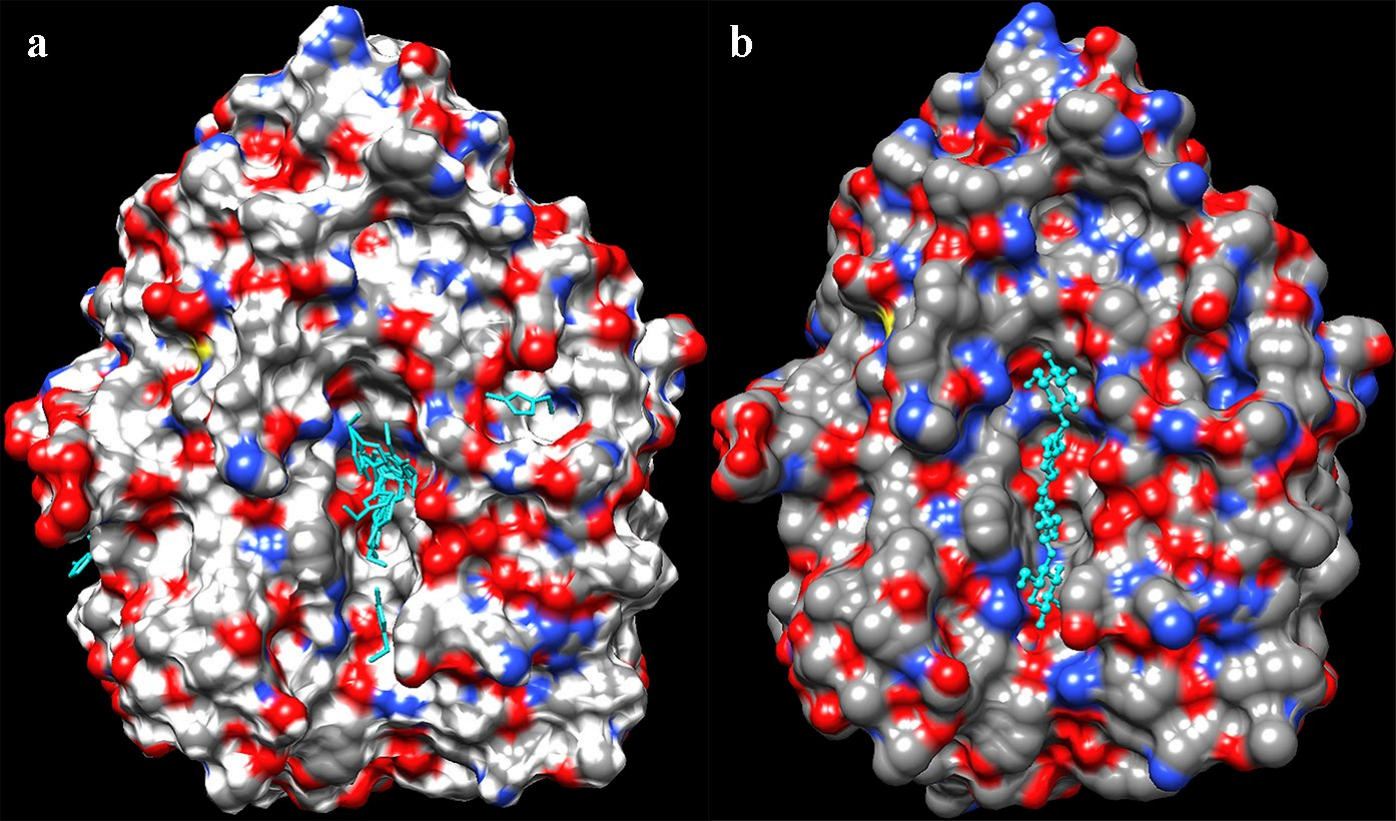
The potential [EMIM]^+^ cation binding sites (**a**), and the cellotetraose substrate molecule (**b**) shown on the surface of *Pyrococcus horikoschii* endoglucanase (PhEG). Cellotetraose is from 3QHM. Ligands are shown in cyan

Molecular docking with [DBNH]^+^, [DBUH]^+^, [EMIM]^+^, [mDBN]^+^ and [TMGH]^+^ was performed to correlate the overall binding behaviour among the observed, about 250 binding poses to the enzyme activity in ILs (Supplementary Table 1). The IL cations are trapped at the active site canyon of the enzyme in the same areas where the substrate binds. The binding areas for the [EMIM]^+^ cation and the cellotetraose substrate are shown in Fig. 8. The figure indicates how the IL cations are trapped onto the active site canyon of the enzyme in the same areas where the substrate binds. The tetrameric substrate shows the key binding areas for the substrate, which typically is much longer than four sugar units. Therefore, the actual substrate that replaces ionic liquid cations from the active site may bind to the enzyme in a larger area than where the IL cations are detected to bind with the observed binding energies (see Supplementary Table 1). Basically, only a few cations can bind concurrently to the active site, and they largely fill it (Supplementary Fig. 9). Other sites for binding of the cation are also observed outside the enzyme active site (Fig. 8). The location and interaction pattern of the highest energy binder of the [EMIM]^+^ cation in a pocket above the catalytic residues of PhEG are shown in Supplementary Fig. 10. The positive charge of [EMIM]^+^ is located close to the catalytic glutamate side chains of PhEG, as was reported earlier for this enzyme (Jaeger et al. 2015), and for other endoglucanases and xylanases (Hebal et al. 2021). The differences in the potential cation binding areas in the active site among the five IL cations are shown in Supplementary Fig. 11. The [EMIM]^+^ cation shows the smallest potential binding area, which reflects its lower inhibition.

In our study, docking with IL anions (acetate, propionate, butyrate and guaiacolate) indicates that as expected the anion with a larger hydrophobic group is better able to fill the PhEG active site canyon (Supplementary Fig. 12), as has also been shown for GH10 xylanase TfXYN10A (Hebal et al. 2020). The difference is that, in PhEG, the anions bind at sites all over the active site, whereas there is a separate anion binding site at the end of the active site cleft in TfXYN10A (Hebal et al. 2020). The greater competitive inhibition of PhEG may be explained by the stronger binding of cations to PhEG (-8.06 to -8.40 kcal/mol) compared to TfXYN10A (− 6.4 to − 7.0 kcal/mol) (Hebal et al. 2020). Moreover, in PhEG, the cations show a greater binding energy compared to the anions (− 6.4 to − 7.1), thereby indicating that cation binding to the active site could be stronger than the binding of anions. In contrast, similar binding energies were observed for the same cations (− 6.4 to − 7.0 kcal/mol) and anions (− 6.48 to − 7.65 kcal/mol) in TfXYN10A xylanase (Hebal et al. 2020).

The ring structures of inhibiting cations ([DBUH]^+^, [DBUH]^+^ and [mDBN]^+^ (see Supplementary Fig. 8) are likely to bind to the same active site positions as the sugar ring structures of the substrate (Chawachart et al. 2014; Summers et al. 2020). Reasons why the [EMIM]^+^ cation is more enzyme friendly compared to the other studied IL molecules could be because it lacks a double ring structure, it is a basic cation, and is not too hydrophobic (see Supplementary Fig. 8). Our docking results indicate that binding of several IL molecules simultaneously onto the active site contributes to the overall effect on enzyme activity, and that there are significant differences in the binding profile between the various IL molecules and between the different enzymes.

## Conclusions

Although the different studies suggest a quite similar general inhibition mechanism for the different enzymes, there exists great variation in the effects of ILs between the enzymes. Superbase ILs can either inhibit or activate PhEG, depending on conditions and the nature of the substrate. The activation of PhEG, in larger extent than TfXYN10A, seems to be the main difference explaining the greater tolerance of this endoglucanase to these ILs. Furthermore, the greater tolerance displayed by PhEG to many ILs at elevated substrate concentrations indicates that it could be successfully used in industrial, high substrate conditions with a range of ILs. Differences in the binding of ILs to the active sites of PhEG and TfXYN10A appears to determine the differing effect of ILs. These findings could assist in the planning of rational approaches to the design of new enzyme friendly ILs and to the engineering of new IL-tolerant enzymes. The success achieved to date in improving the tolerance of cellulases to ILs in some of the rational approaches is promising (Lee et al. 2017; Pramanik et al. 2021; Summers et al. 2020). Important aspects to consider in planning are that enzymes differ in their response to various ILs and that different substrates may behave differently in IL solutions.

## Supplementary Information

Below is the link to the electronic supplementary material.

Supplementary file1 (DOC 5449 KB)
