# Supplementary material for: Enhanced activity of hyperthermostable Pyrococcus horikoshii endoglucanase in superbase ionic liquids
Source: Biotechnol Lett. 2022 Jun 28;44(8):961–74. doi: 10.1007/s10529-022-03268-5 (PMC9356960; doi:10.1007/s10529-022-03268-5)
Supplement: Supplementary file 1 — Supplementary file1 (DOC 5449 KB) [file 10529_2022_3268_MOESM1_ESM.doc]

**Biotechnology letters**

**Supplementary information**

**Enhanced activity of hyperthermostable *Pyrococcus horikoshii* endoglucanase in superbase ionic liquids**

Hakim Hebal^1,2^, Joonas Hämäläinen^3^, Laura Makkonen^4^, Alistair W. T. King^5^, Ilkka Kilpeläinen^5^, Sandip Bankar^4^, Nawel Boucherba^1^ and Ossi Turunen^6*^

1. Laboratoire de Microbiologie Appliquée (LMA), Faculté des Sciences de La Nature et de La Vie (FSNV), Université de Bejaia, Bejaia, Algeria.
2. Faculty of Exact Sciences and Sciences of Nature and Life, Department of Biology, Mohamed Khider University of Biskra, Biskra, Algeria
3. St1 Oy, Firdonkatu 2, PL 68, 00521 Helsinki
4. Department of Bioproducts and Biosystems, School of Chemical engineering, FI-00076 Aalto University, Finland
5. Department of Chemistry, FI-00014 University of Helsinki, Finland
6. Faculty of Science and Forestry, School of Forest Sciences, University of Eastern Finland, FI-80101 Joensuu, Finland

*Corresponding author. E-mail: ossi.turunen@uef.fi; Phone: +358503425151

**Experimental graphs**


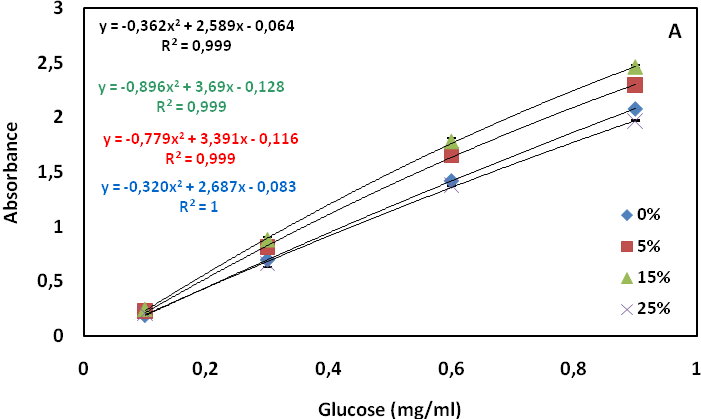


**Figure 1A.** Glucose calibration graphs in the presence of [DBNH]OAc.


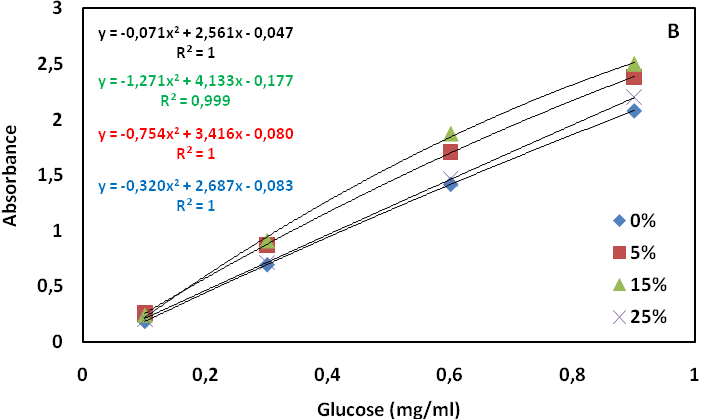


**Figure 1B.** Glucose calibration graphs in the presence of [DBNH]CO_2_Et.


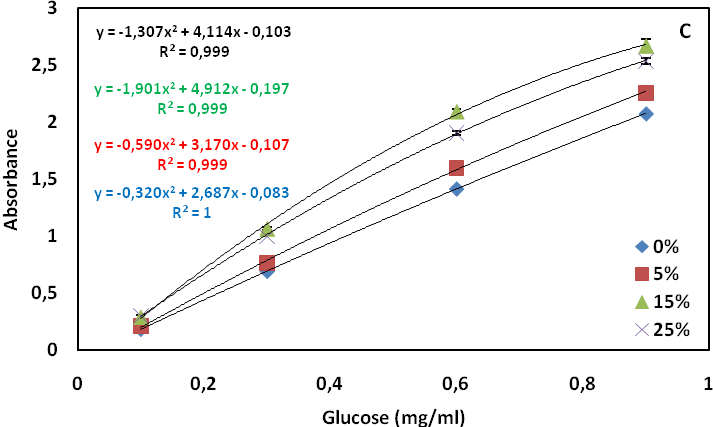


**Figure 1C.** Glucose calibration graphs in the presence of [DBUH]OAc.


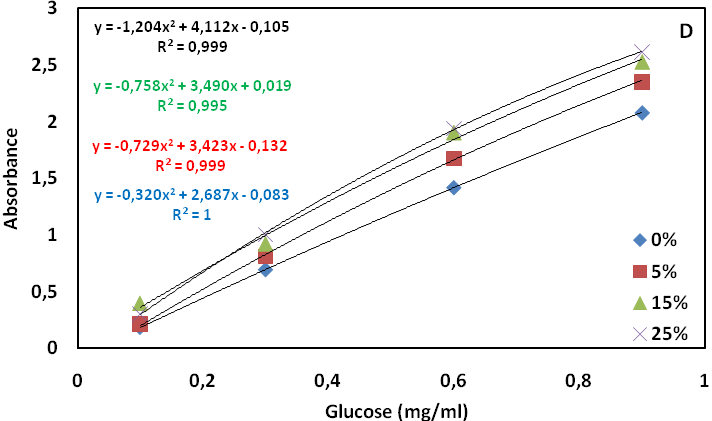


**Figure 1D.** Glucose calibration graphs in the presence of [DBUH]CO_2_Et.


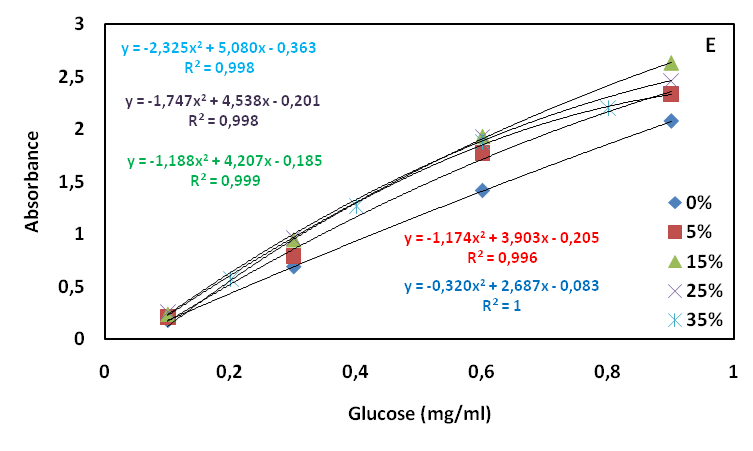


**Figure 1E.** Glucose calibration graphs in the presence of [EMIM]OAc.


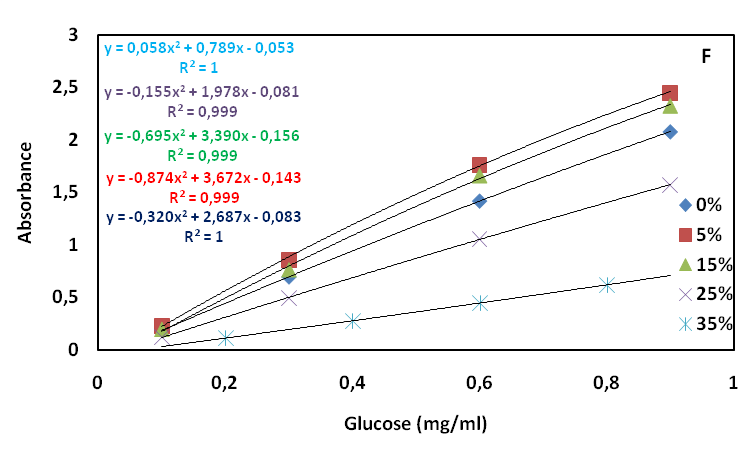


**Figure 1F.** Glucose calibration graphs in the presence of [mDBN]Me_2_PO_4_.


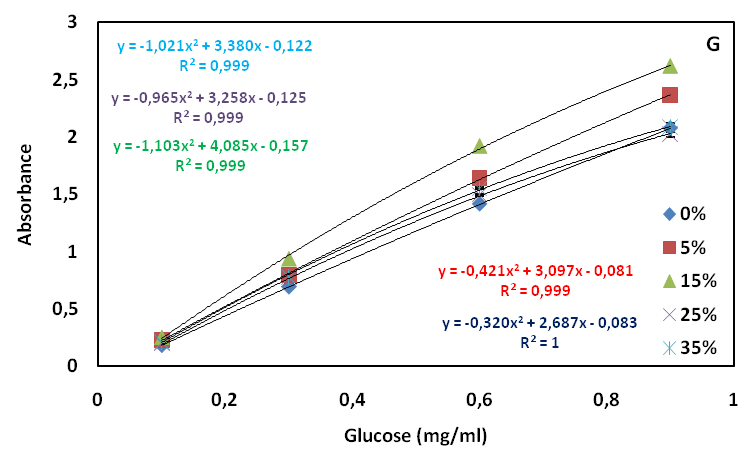


**Figure 1G.** Glucose calibration graphs in the presence of [TMGH]OAc.


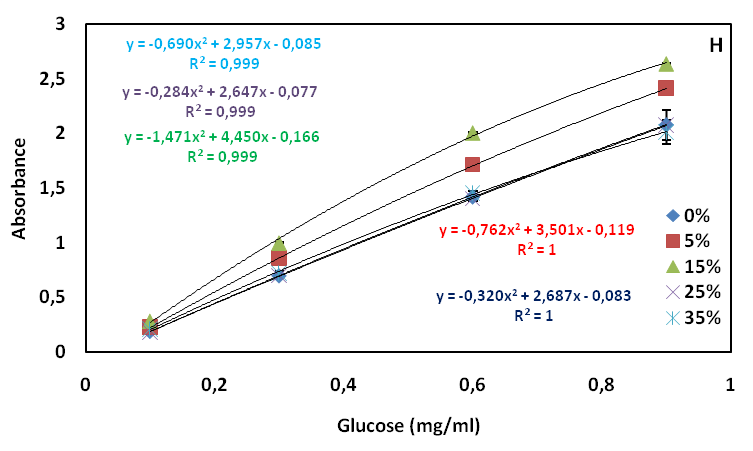


**Figure 1H.** Glucose calibration graphs in the presence of [TMGH]CO_2_Et.


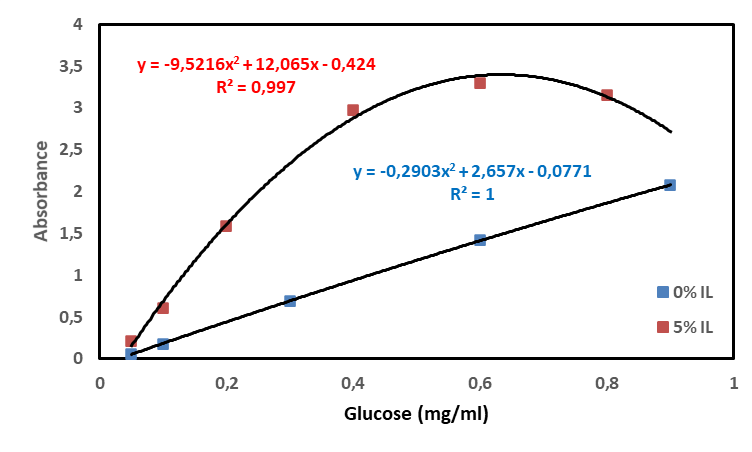


**Figure 1I.** Glucose calibration graph in the presence of 5% [DBNH]guaiacolate.


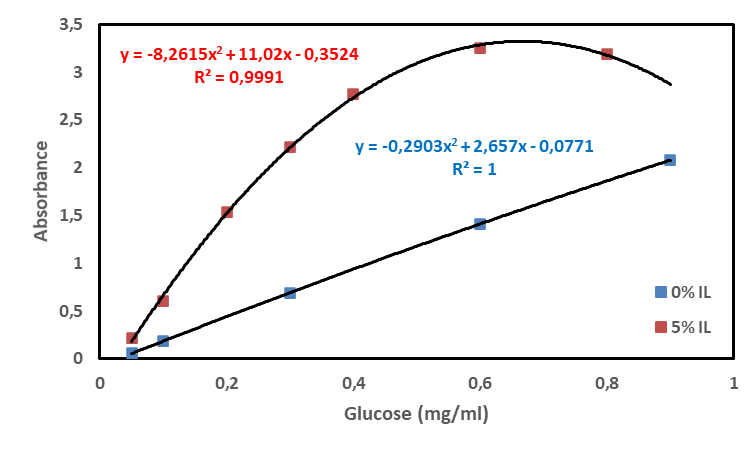


**Figure 1J.** Glucose calibration graph in the presence of 5% [DBUH]guaiacolate.


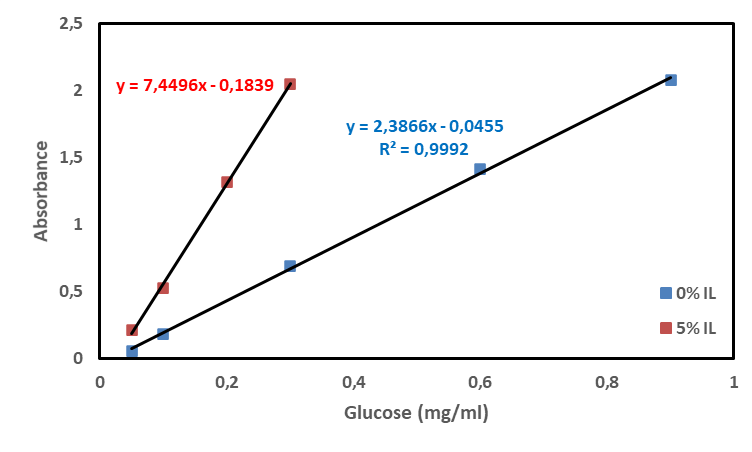


**Figure 1K.** Glucose calibration graph in the presence of 5% [TMGH]guaiacolate.

| **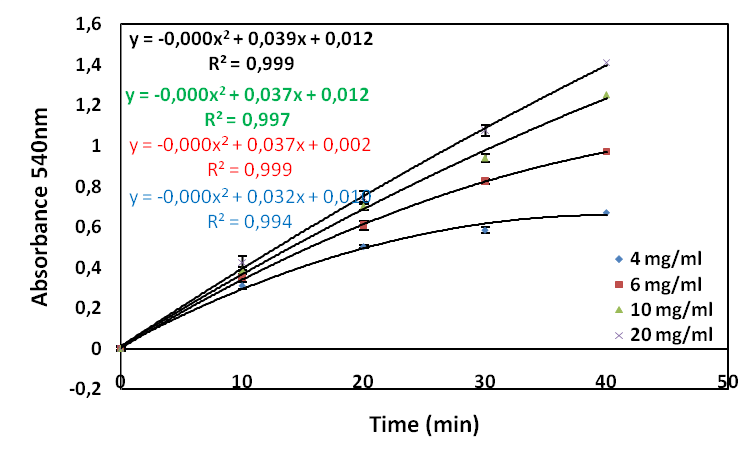**  **A** |
| --- |
| **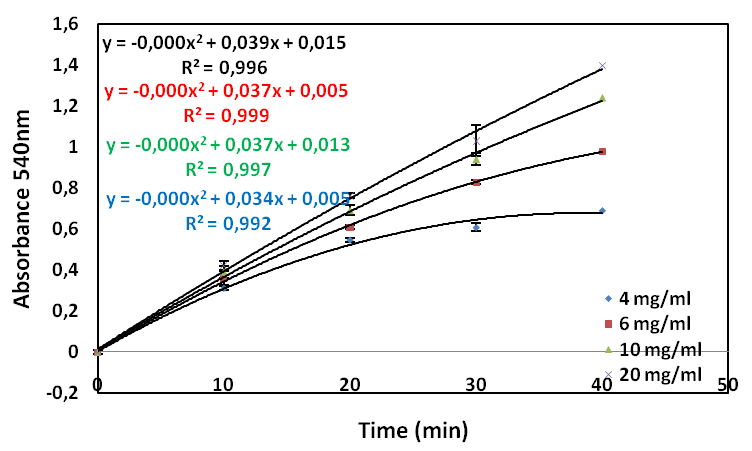** |
| **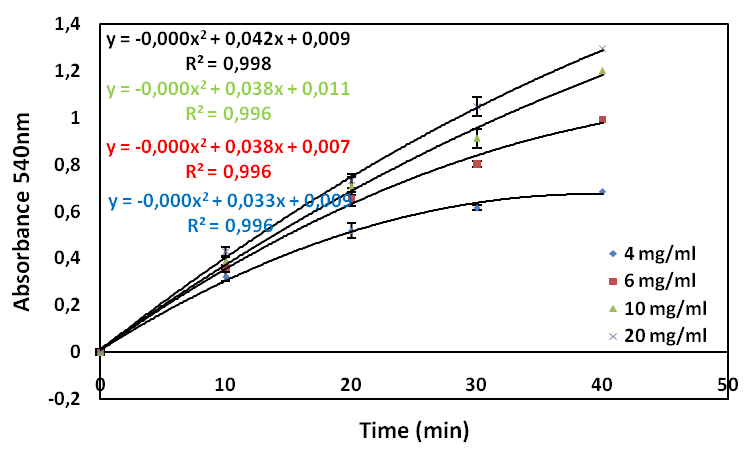** |

**Figure 2A**. Time-dependent hydrolysis by *Pyrococcus horikoshii* endoglucanase (PhEG) in the absence of ionic liquids (three independent experiments).

| 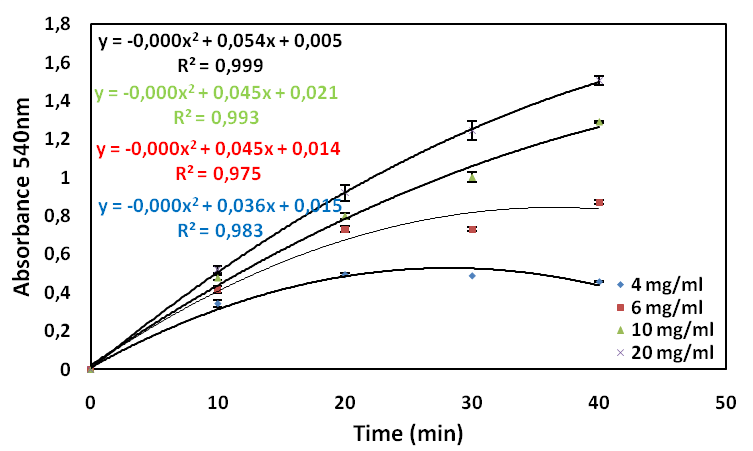  **B** |
| --- |
| 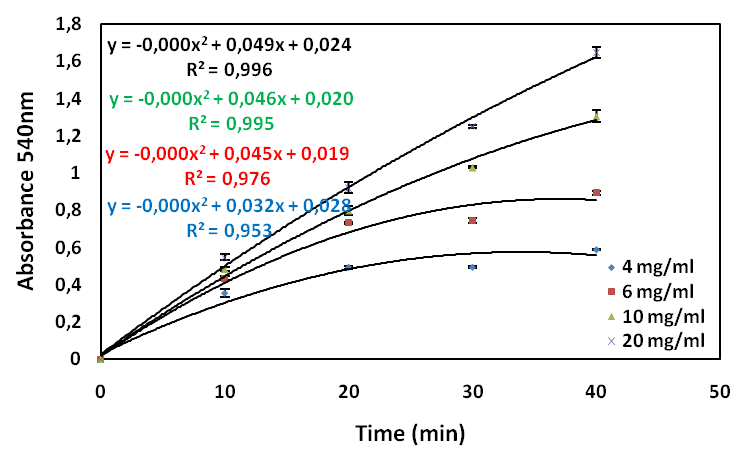 |
| 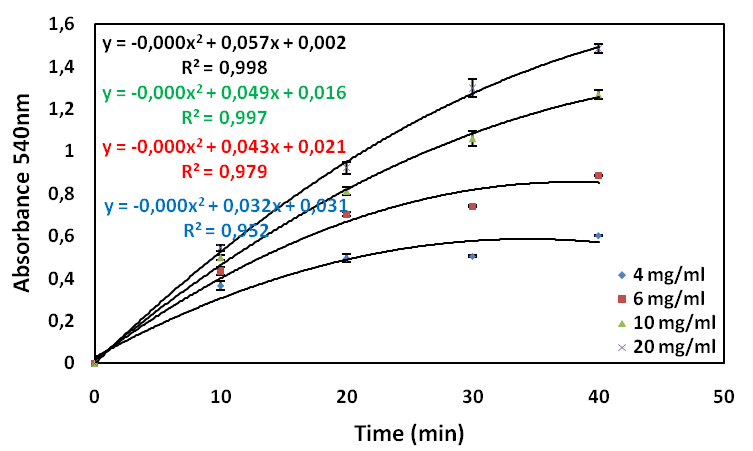 |

**Figure 2B.** Time-dependent hydrolysis by PhEG in the presence of [DBNH]OAc (three independent experiments).

| 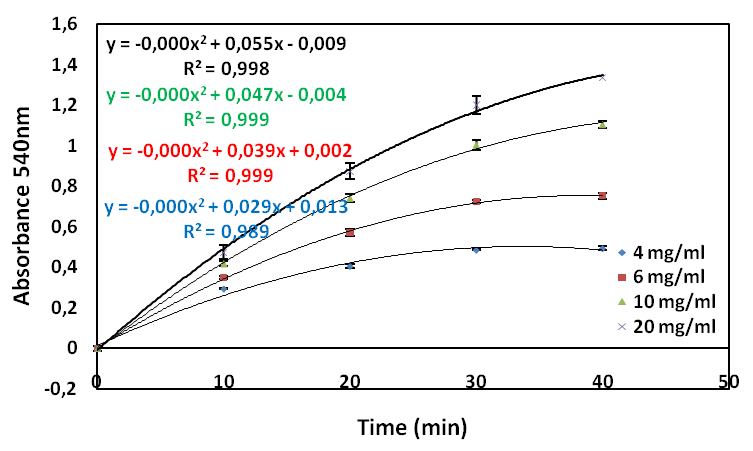  **C** |
| --- |
| 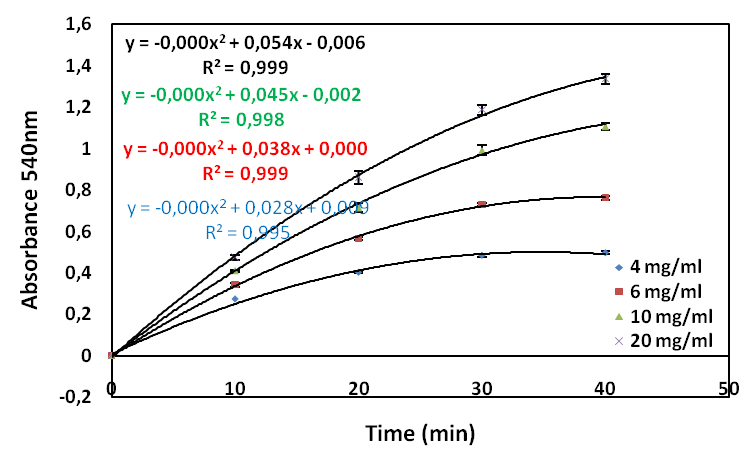 |
| 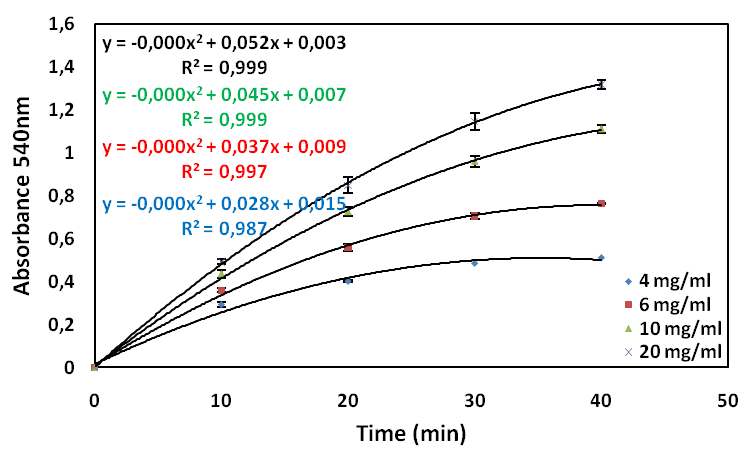 |

**Figure 2C.** Time-dependent hydrolysis by PhEG in the presence of [DBNH]CO_2_Et (three independent experiments).

| 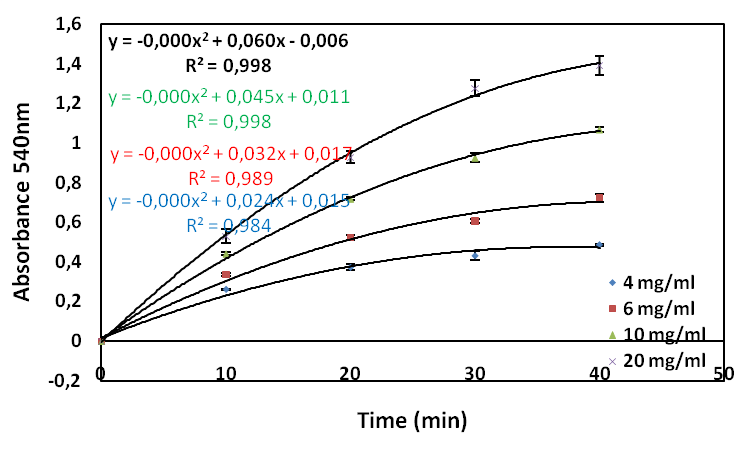  **D** |
| --- |
| 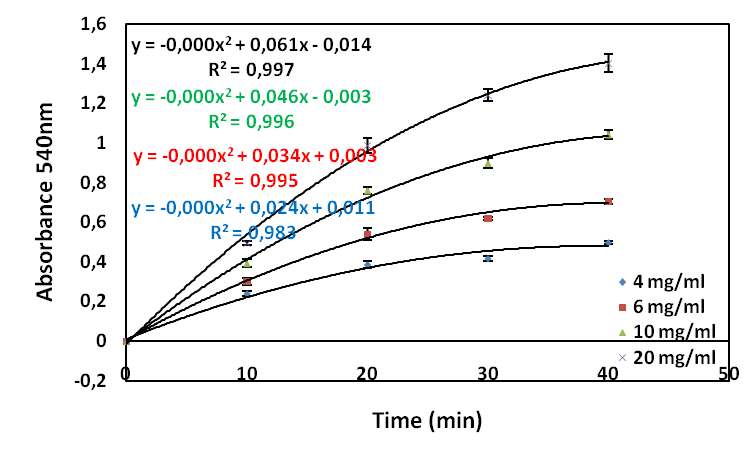 |
| 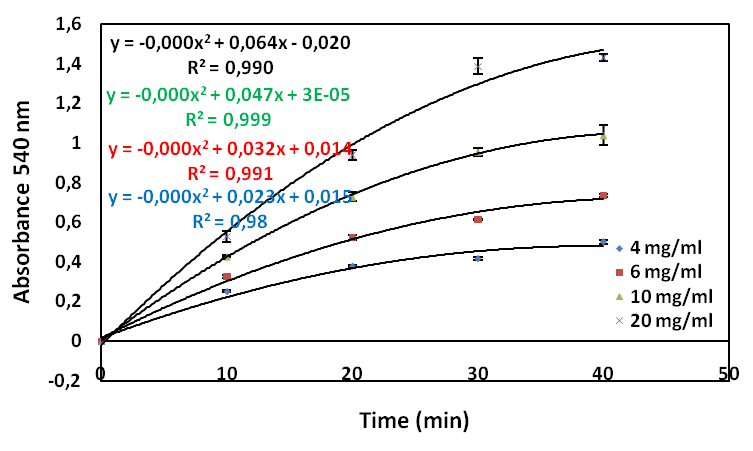 |

**Figure 2D.** Time-dependent hydrolysis by PhEG in the presence of [DBUH]OAc (three independent experiments).

| 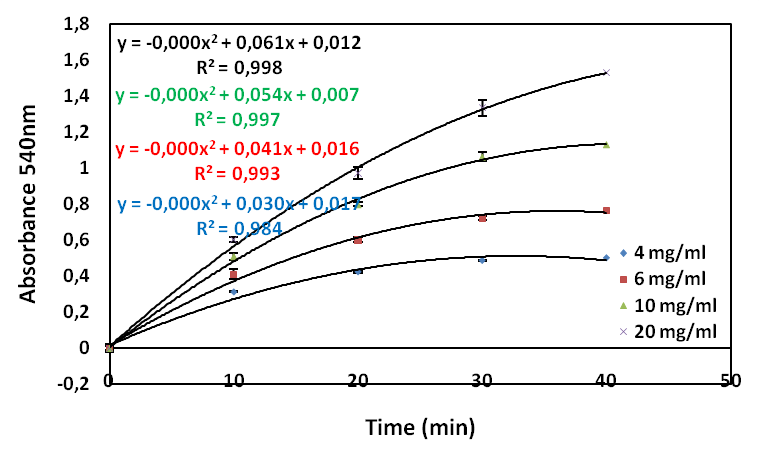  **E** |
| --- |
| 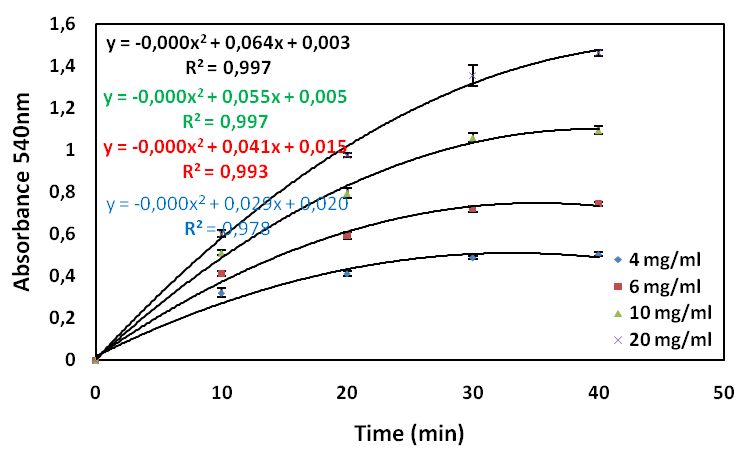 |
| 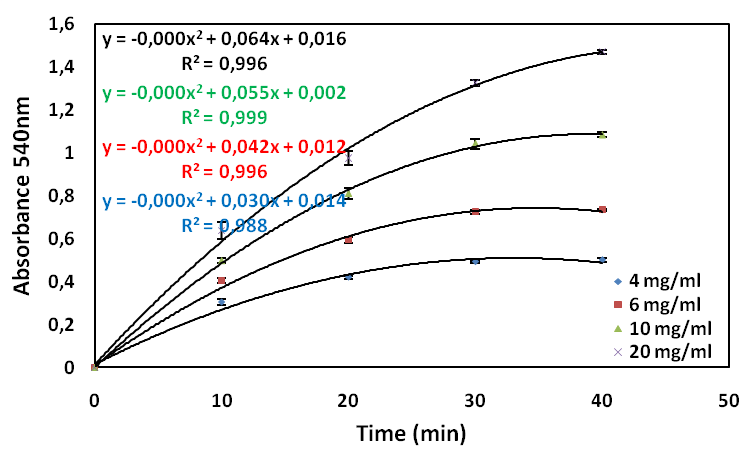 |

**Figure 2E.** Time-dependent hydrolysis by PhEG in the presence of [DBUH]CO_2_Et (three independent experiments).

| 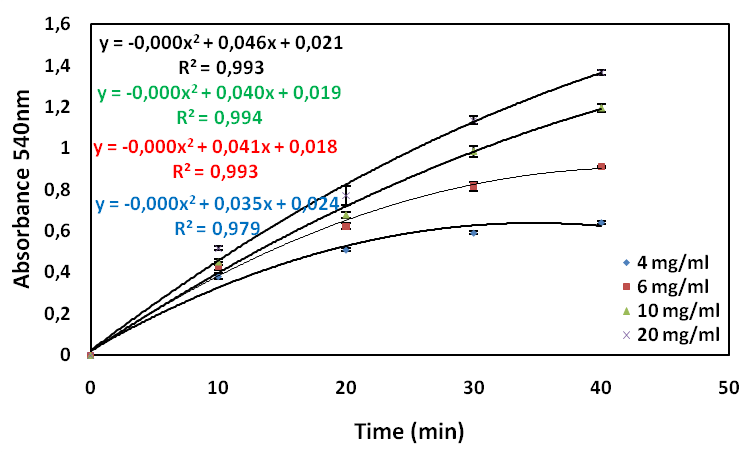  **F** |
| --- |
|  |
| **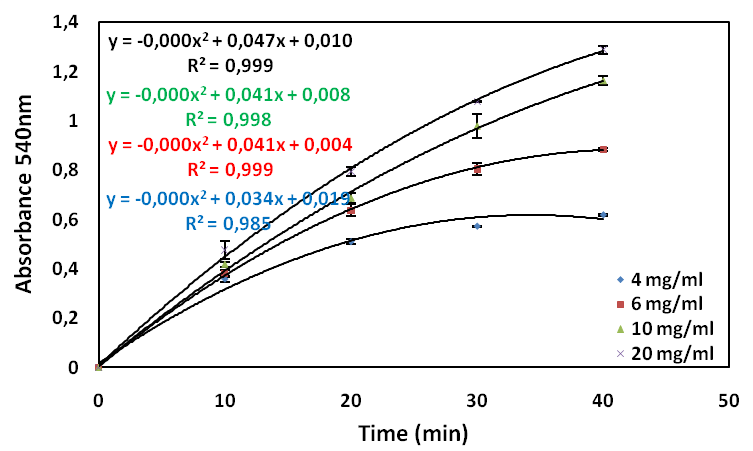**  **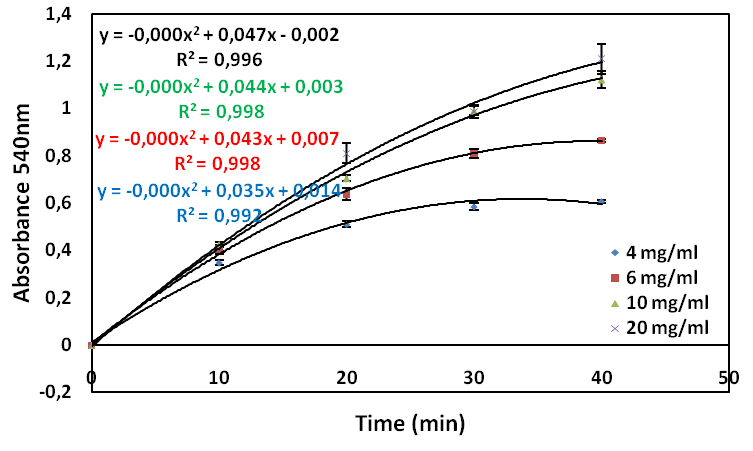** |

**Figure 2F.** Time-dependent hydrolysis by PhEG in the presence of [EMIM]OAc (three independent experiments).

| 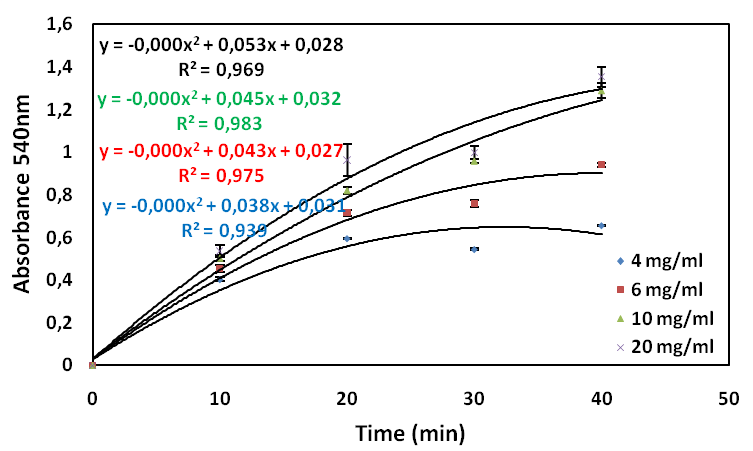  **G** |
| --- |
| **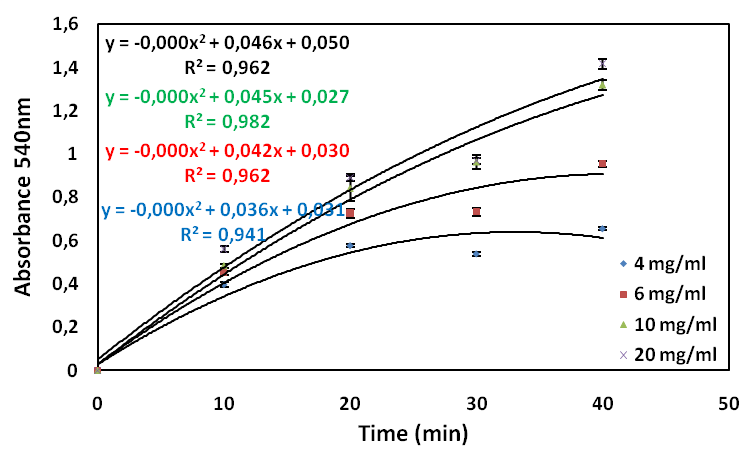** |
| **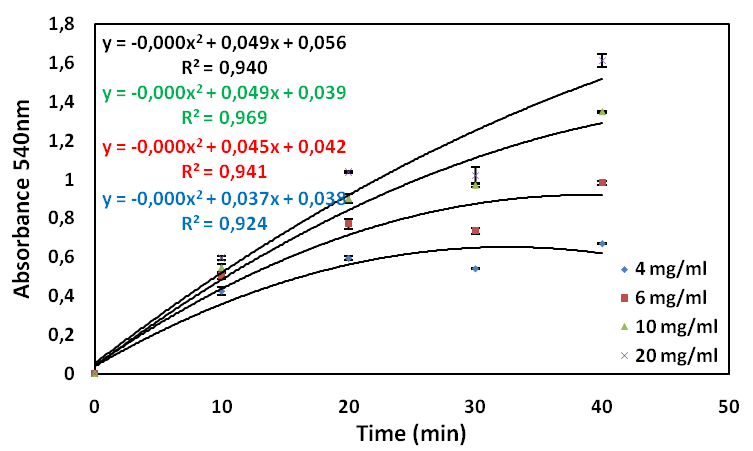** |

**Figure 2G.** Time-dependent hydrolysis by PhEG in the presence of [mDBN]Me_2_PO_4_ (three independent experiments).

| 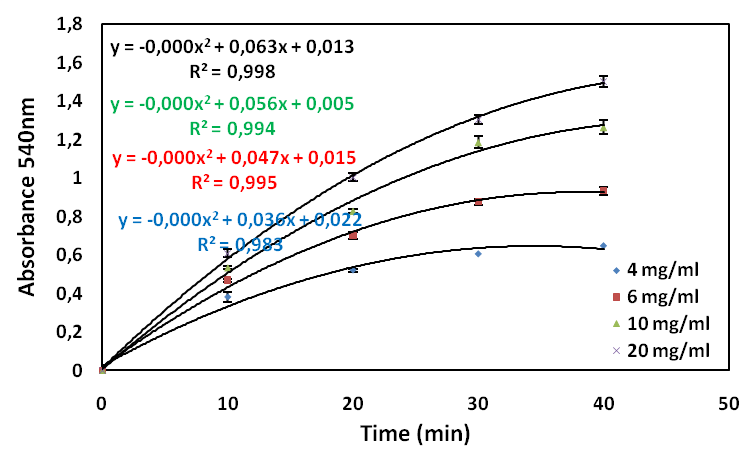  **H** |
| --- |
| **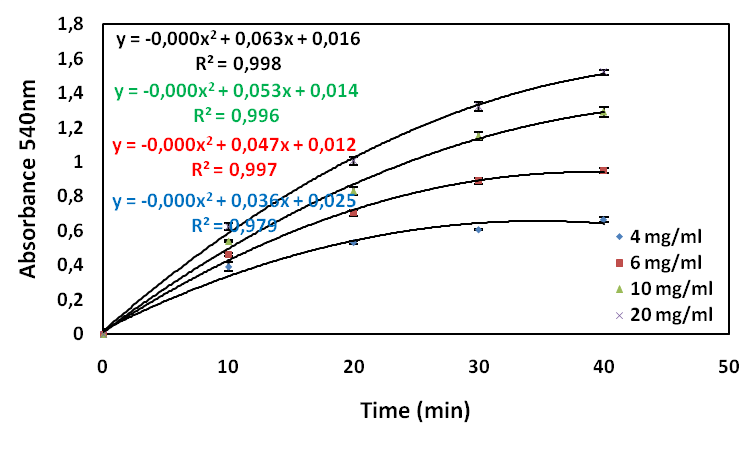** |
| **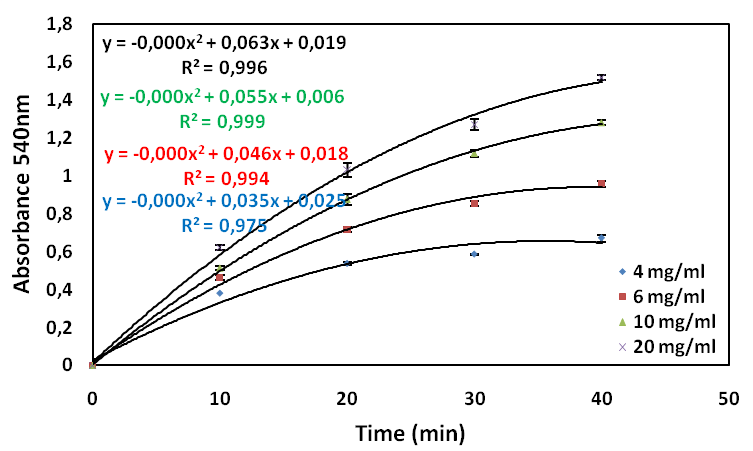** |

**Figure 2H.** Time-dependent hydrolysis by PhEG in the presence of [TMGH]OAc (three independent experiments).

| 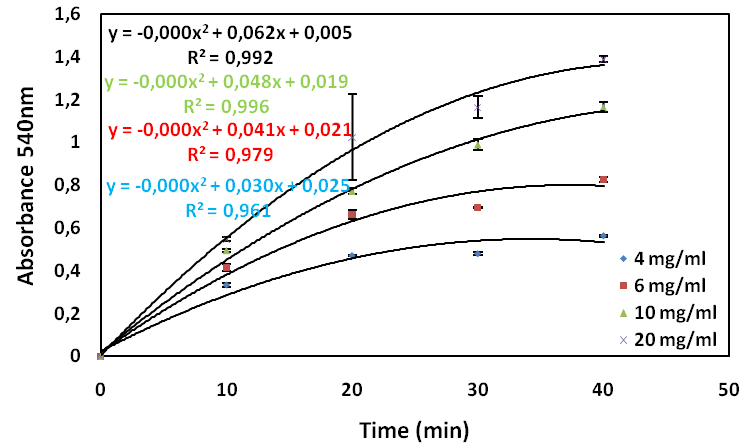  **I** |
| --- |
| **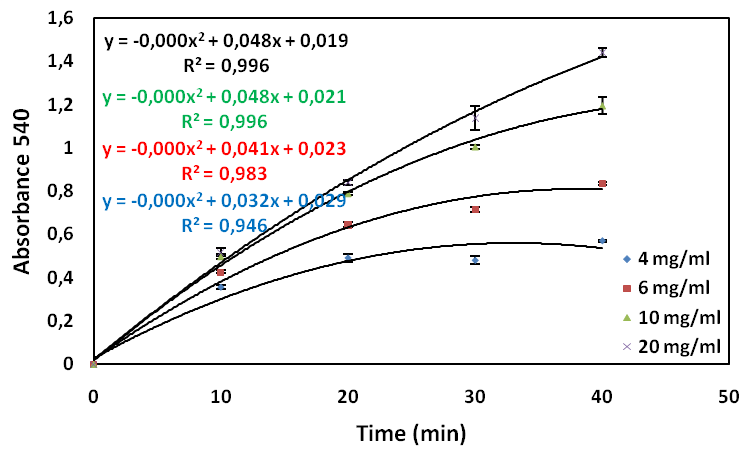** |
| **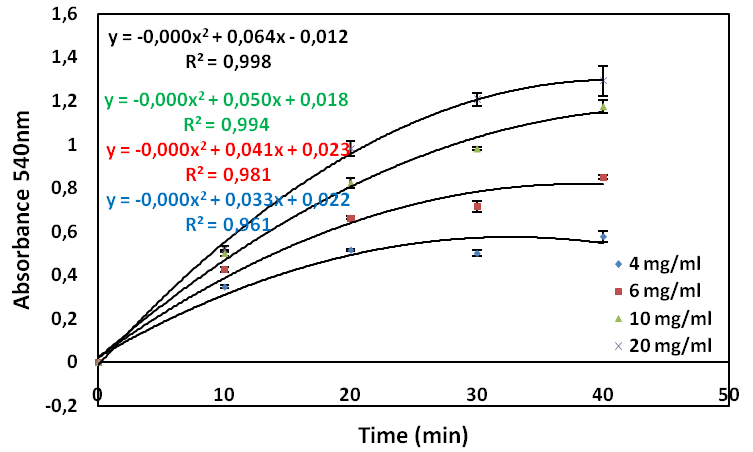** |

**Figure 2I.** Time-dependent hydrolysis by PhEG in the presence of [TMGH]CO_2_Et (three independent experiments).

| 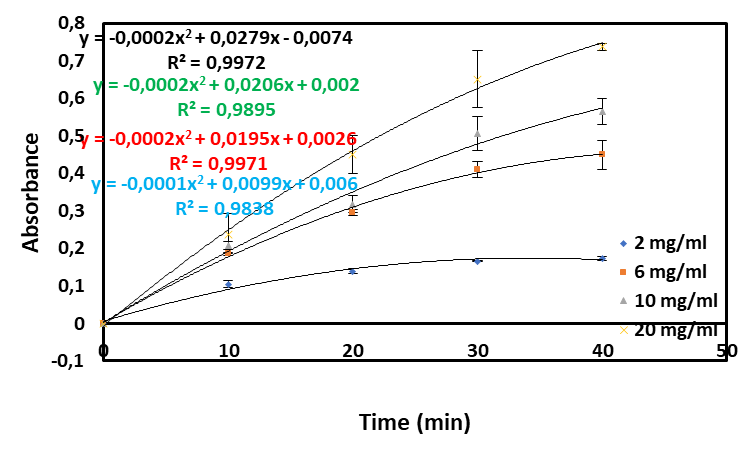  **J**  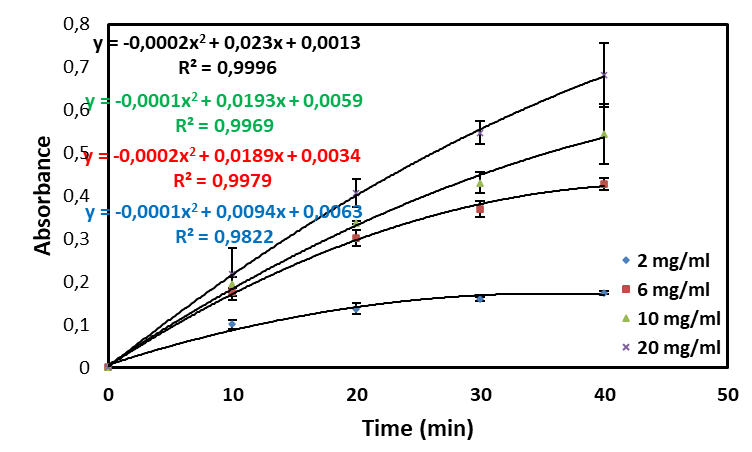 |
| --- |
| 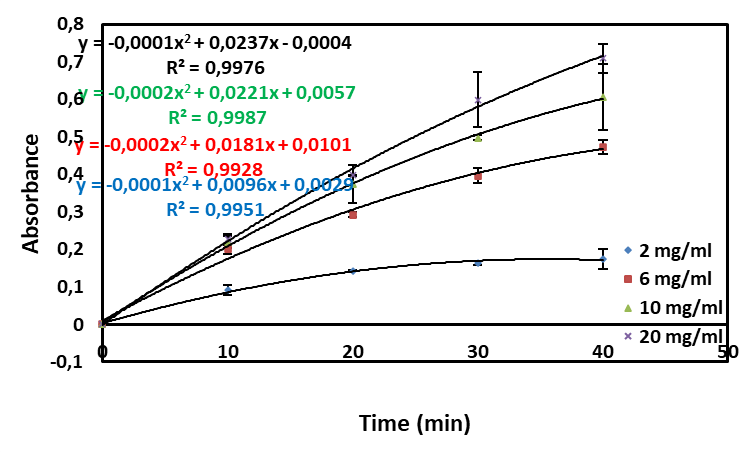 |

**Figure 2J.** Time-dependent hydrolysis by PhEG in the presence of [DBNH]guaiacolate (three independent experiments).

| 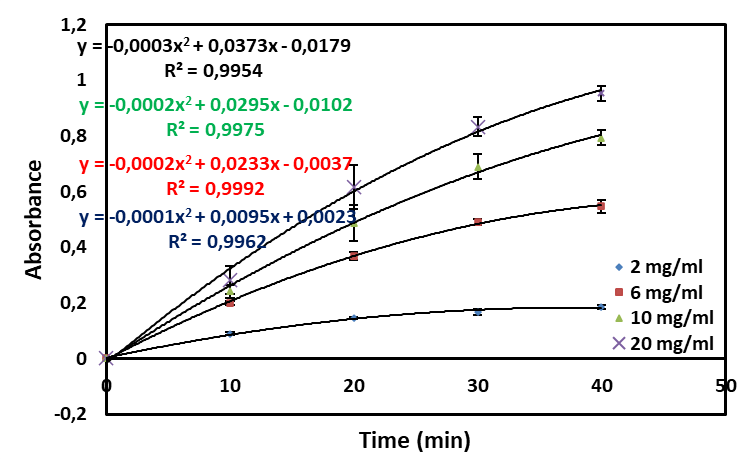  **K**  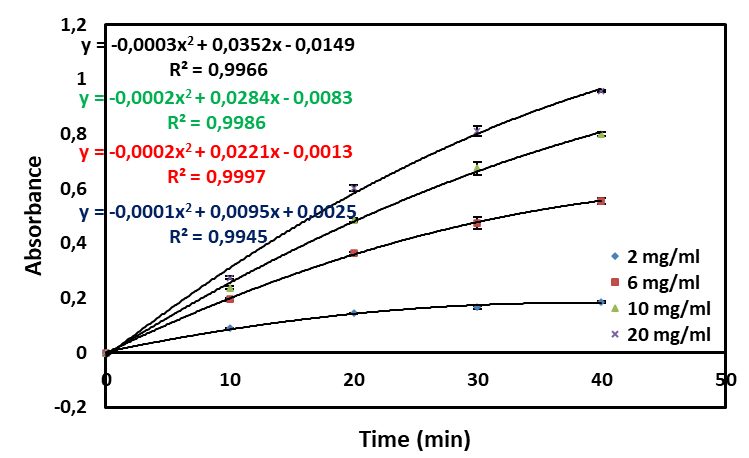 |
| --- |
| 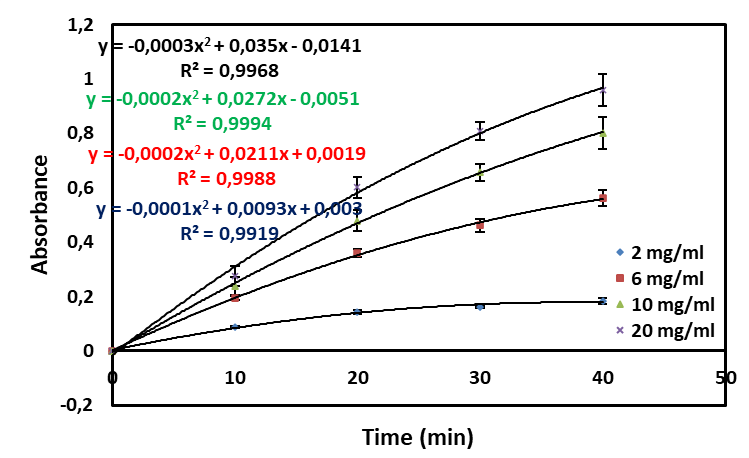 |

**Figure 2K.** Time-dependent hydrolysis by PhEG in the presence of [DBUH]guaiacolate (three independent experiments).

| 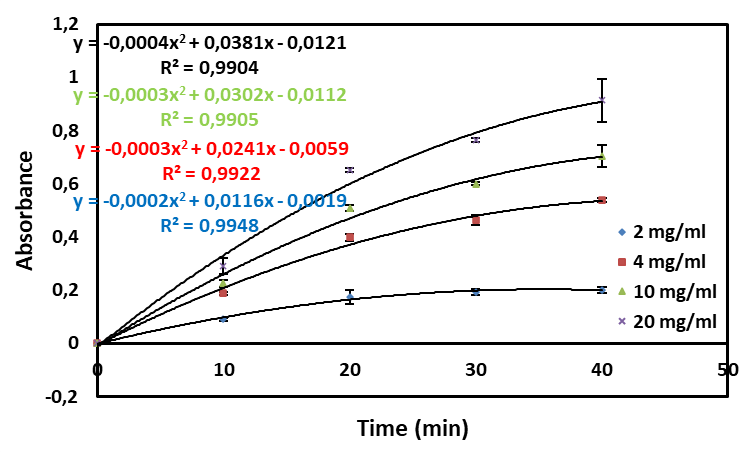  **L**  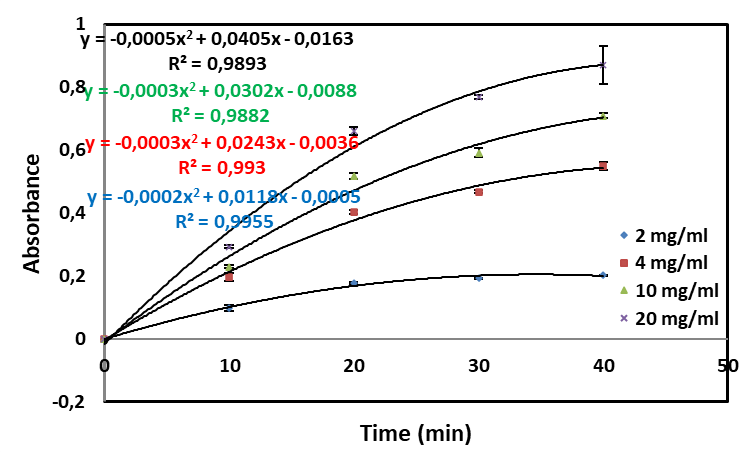 |
| --- |
| 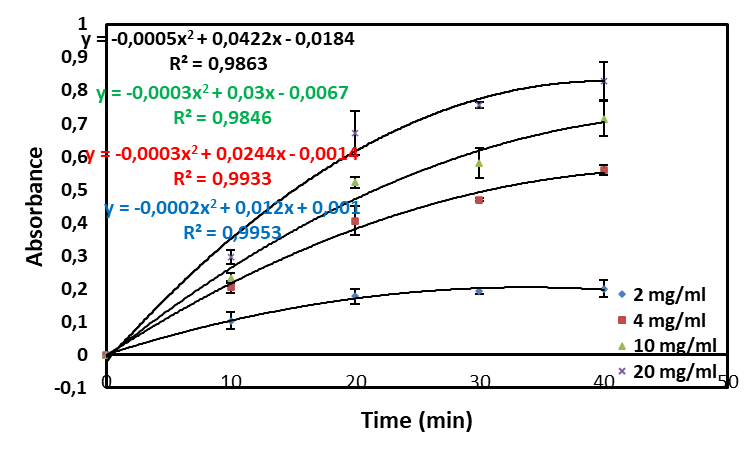 |

**Figure 2L.** Time-dependent hydrolysis by PhEG in the presence of [TMGH]guaiacolate (three independent experiments).

| 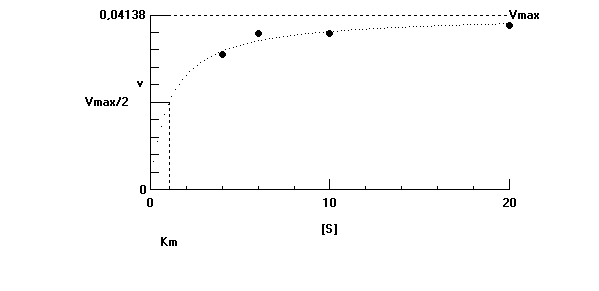  **A** |
| --- |
| 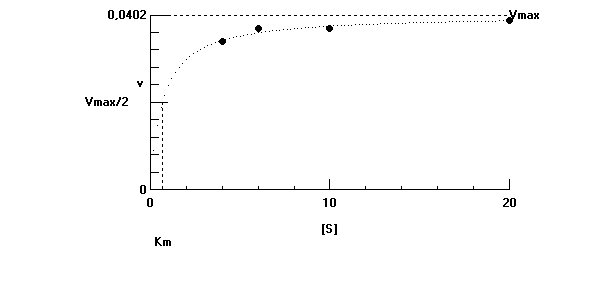 |
| 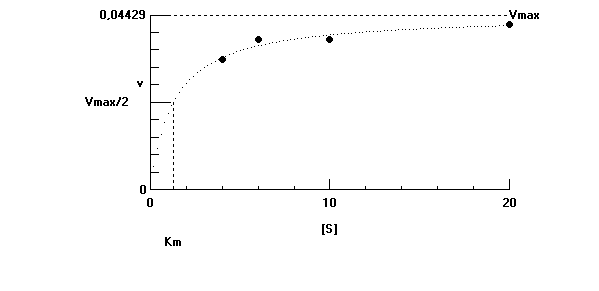 |

Figure 3A. Hyperbolic regression representation of PhEG kinetics in the absence of ionic liquids.

| 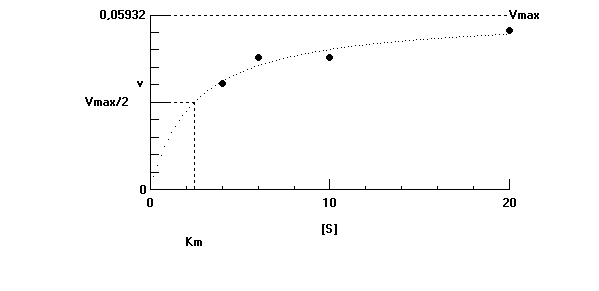  **B** |
| --- |
| 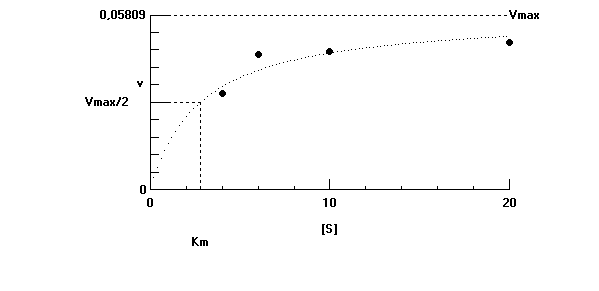 |
| 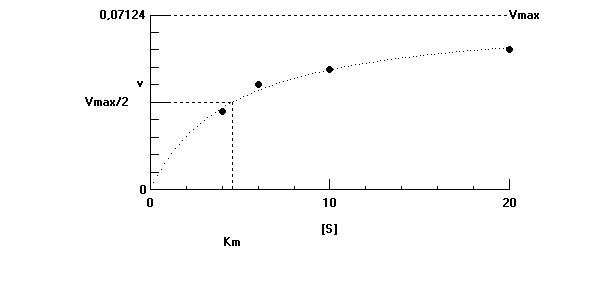 |

**Figure 3B.** Hyperbolic regression representation of PhEG kinetics in the presence of [DBNH]OAc.

| 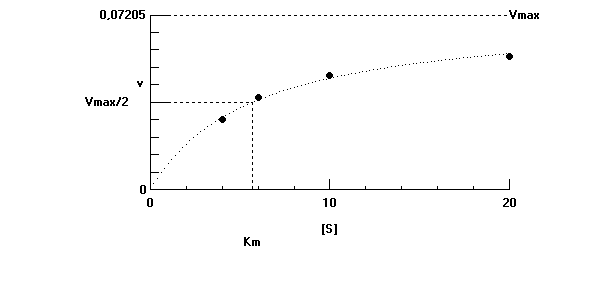  **C** |
| --- |
| 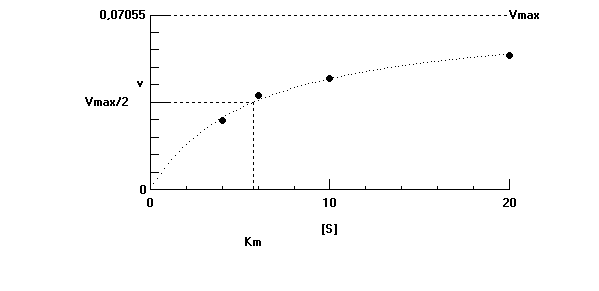 |
| 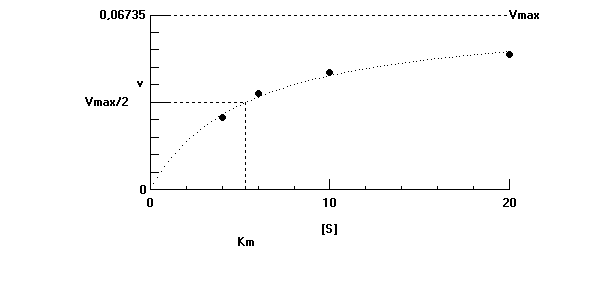 |

Figure 1C. Hyperbolic regression representation of PhEG kinetics in the presence of [DBNH]CO_2_Et.

| 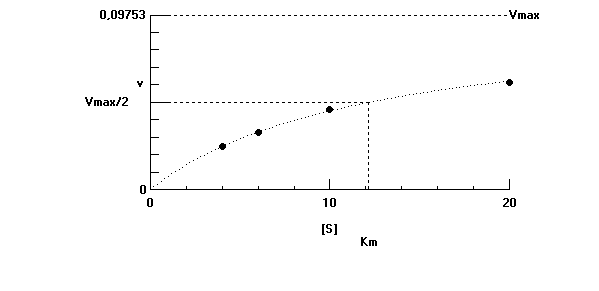  **D** |
| --- |
| 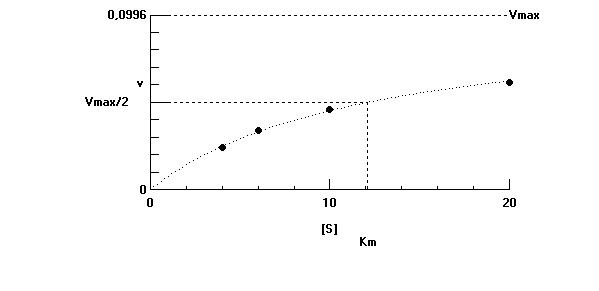 |
| 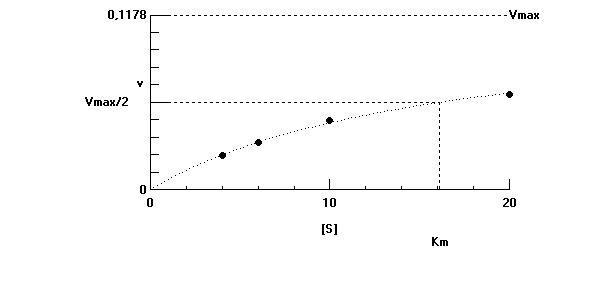 |

Figure 3D. Hyperbolic regression representation of PhEG kinetics in the presence of [DBUH]OAc.

| 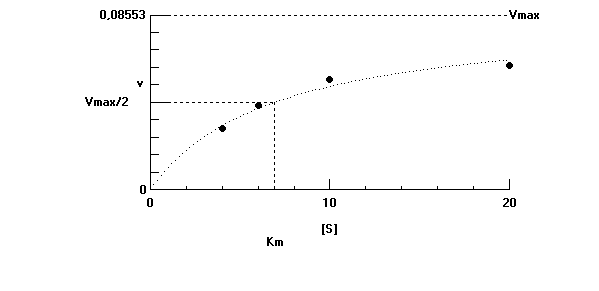  **E** |
| --- |
| 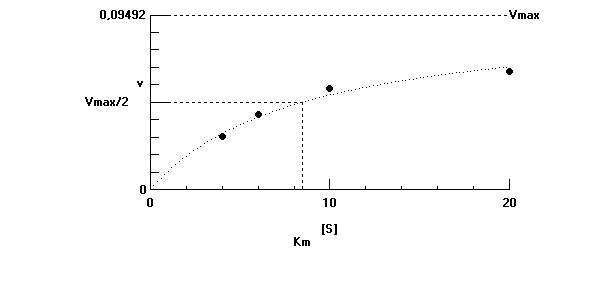 |
| 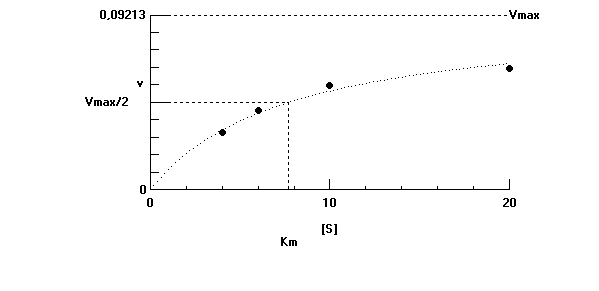 |

Figure 3E. Hyperbolic regression representation of PhEG kinetics in the presence of [DBUH]CO_2_Et.

| 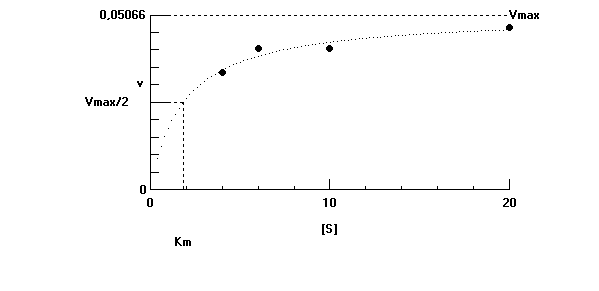  **F** |
| --- |
| 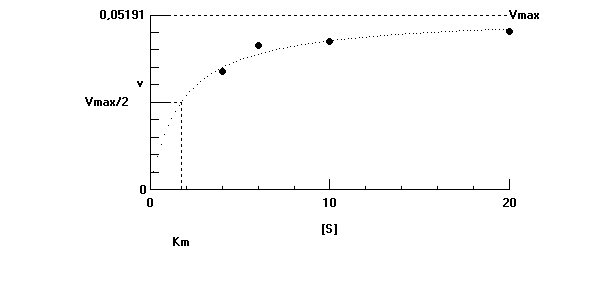 |
| 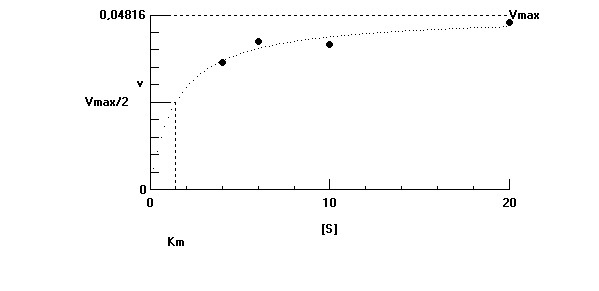 |

Figure 3F. Hyperbolic regression representation of PhEG kinetics in the presence of [EMIM]OAc.

| 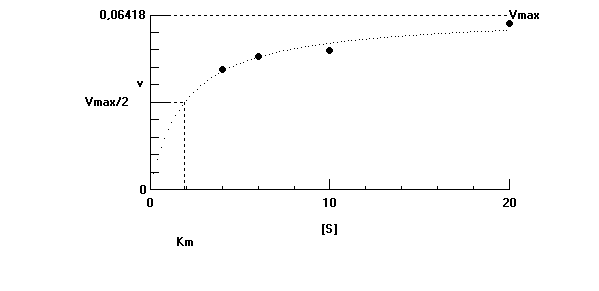  **G** |
| --- |
| 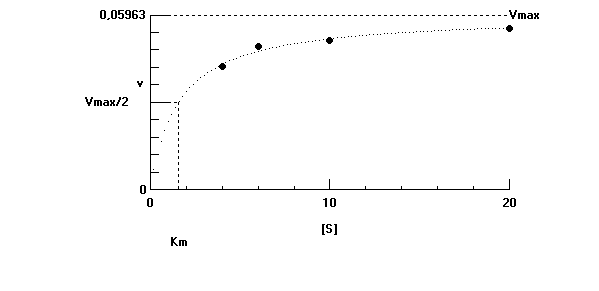 |
| 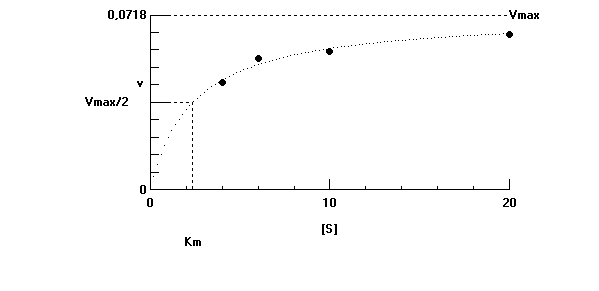 |

Figure 3G. Hyperbolic regression representation of PhEG kinetics in the presence of [mDBN]Me_2_PO_4_.

| 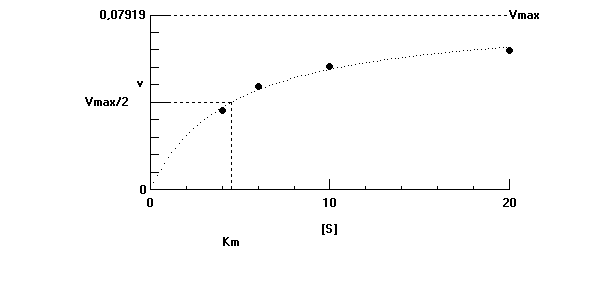  **H** |
| --- |
| 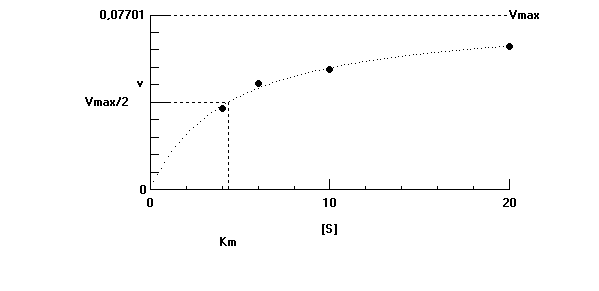 |
| 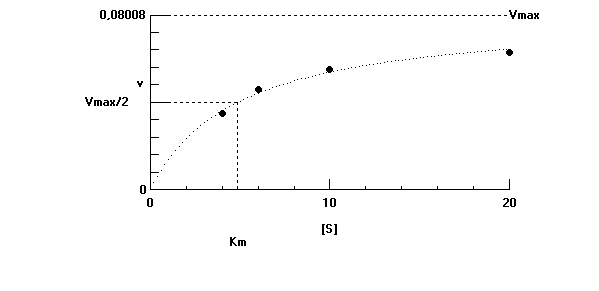 |

Figure 3H. Hyperbolic regression representation of PhEG kinetics in the presence of [TMGH]OAc.

| 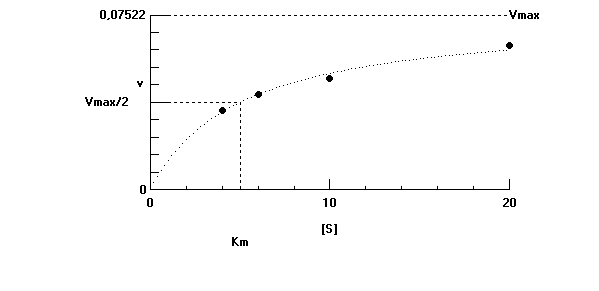  **I** |
| --- |
| 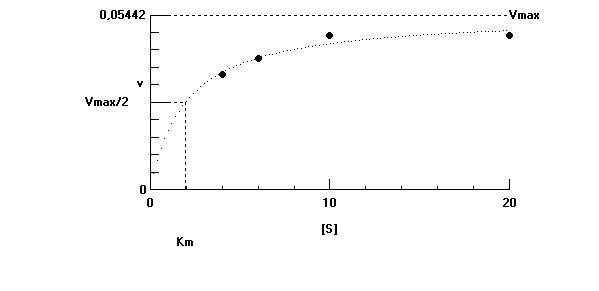 |
| 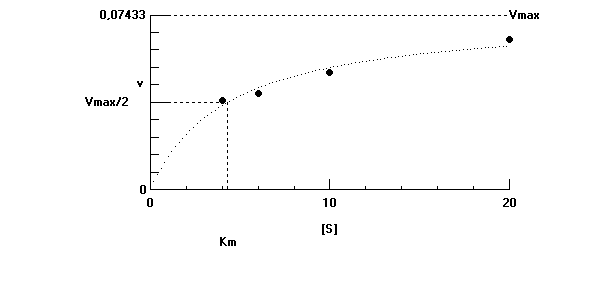 |

**Figure 3I.** Hyperbolic regression representation of PhEG kinetics in the presence of [TMGH]CO_2_Et.

| 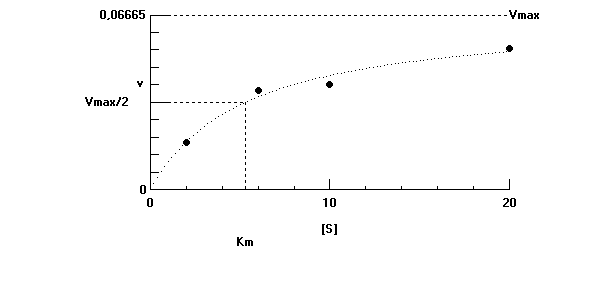  **J** |
| --- |
| 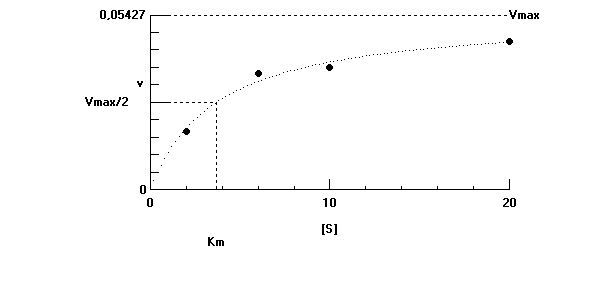 |
| 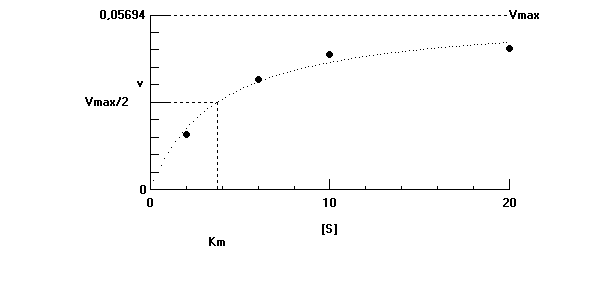 |

**Figure 3J.** Hyperbolic regression representation of PhEG kinetics in the presence of [DBNH]guaiacolate.

| 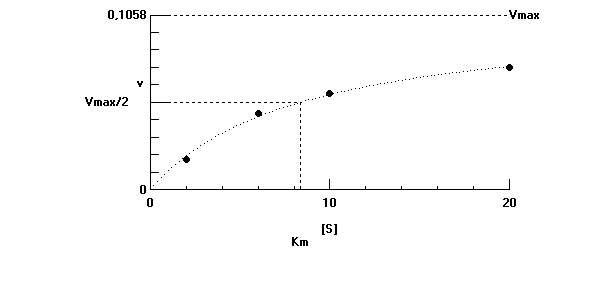  **K** |
| --- |
| 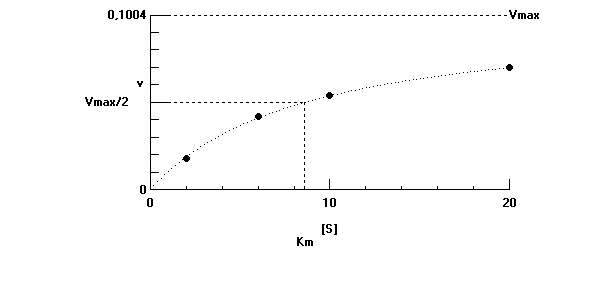 |
| 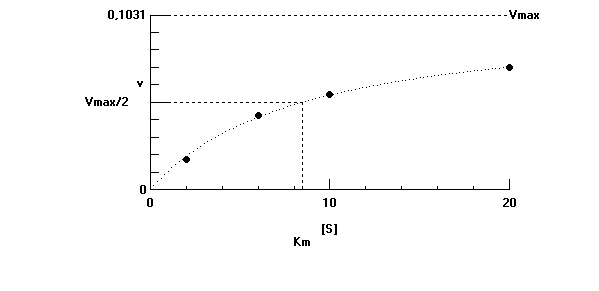 |

**Figure 3K.** Hyperbolic regression representation of PhEG kinetics in the presence of [DBUH]guaiacolate.

| 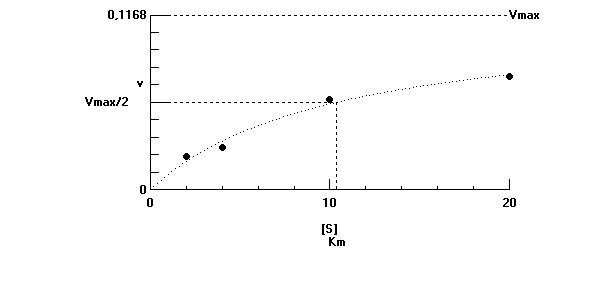  **L** |
| --- |
| 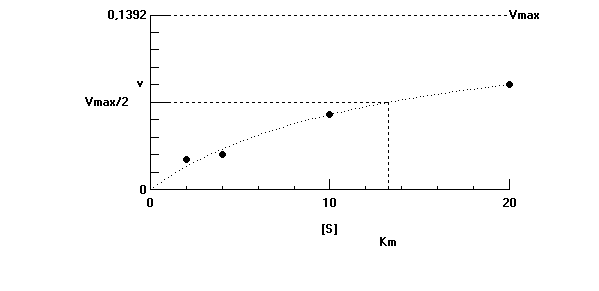 |
| 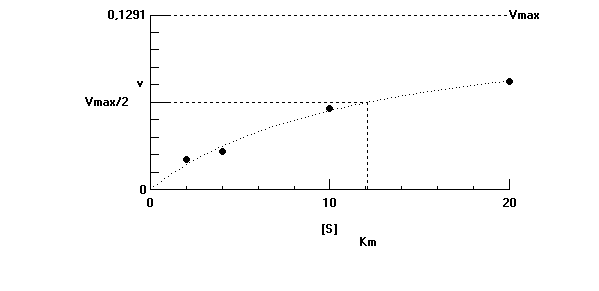 |

**Figure 3L.** Hyperbolic regression representation of PhEG kinetics in the presence of [TMGH]guaiacolate.


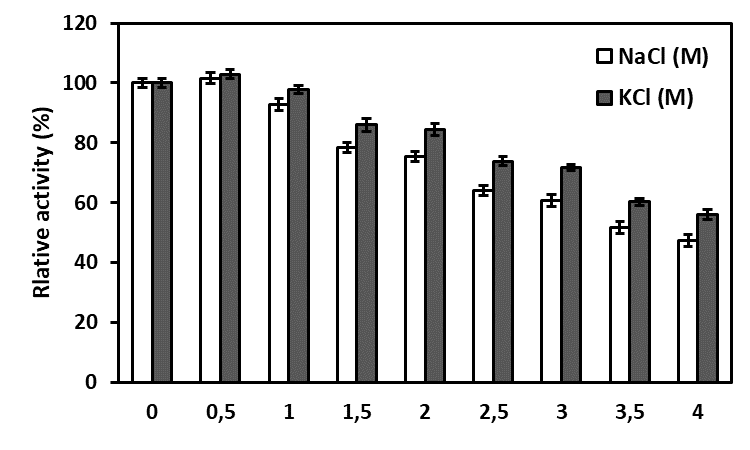


**Figure 4.** Effect of NaCl and KCl on the activity of PhEG.


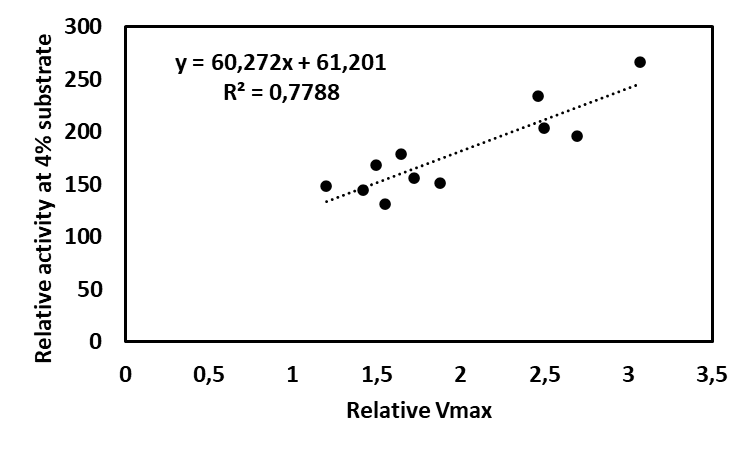


**Figure 5**. Correlation between relative enzyme activity with 4% substrate in the presence of ionic liquids and relative *V*_max_. The relative *V*_max_ values are from the Table 2 in the main text.


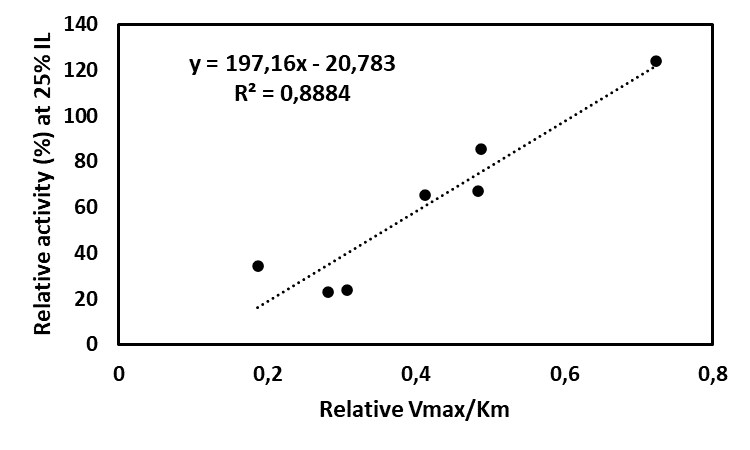


**Figure 6**. Correlation between relative enzyme activity in 25% IL and relative *V*_max_/*K*_m_ in the presence of ionic liquids. The *V*_max_/*K*_m_ values are from the Table 2 in the main text.


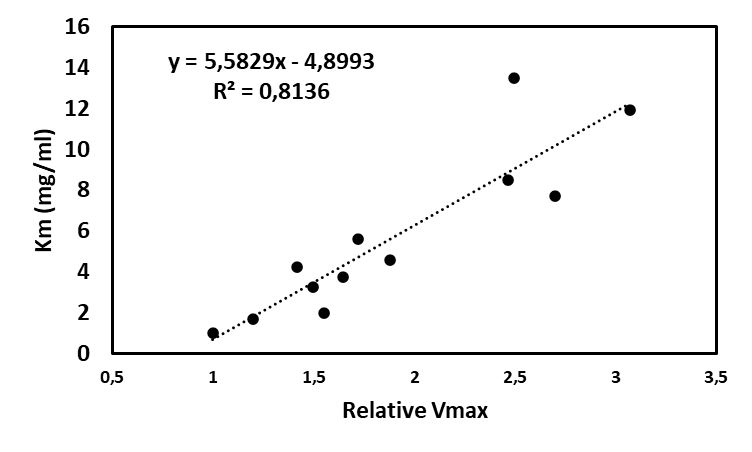


**Figure 7**. Correlation between *K*_m_ and *V*_max_ in the presence of ionic liquids. The *K*_m_ and relative *V*_max_ values are from the Table 2 in the main text. The values without ILs are also included.

**Molecular docking of IL cations and anions to PhEG** **by SwissDock**

The molecular dockings were done by SwissDock with the accurate mode. Flexibility was allowed for the ligand but not for the side chains in all dockings. Other details of docking are described in Hebal et al. (2020). For the structures of the cations used in the study, see Fig. 8. Fig. 9 shows how many IL cations can bind at the same time to the active site. Fig. 10 shows the position for the highest energy binder of the [EMIM]^+^ cation in the active site of PhEG. Fig. 11 and 12 show the cation and anion, respectively, binding site distribution in the active site of PhEG.


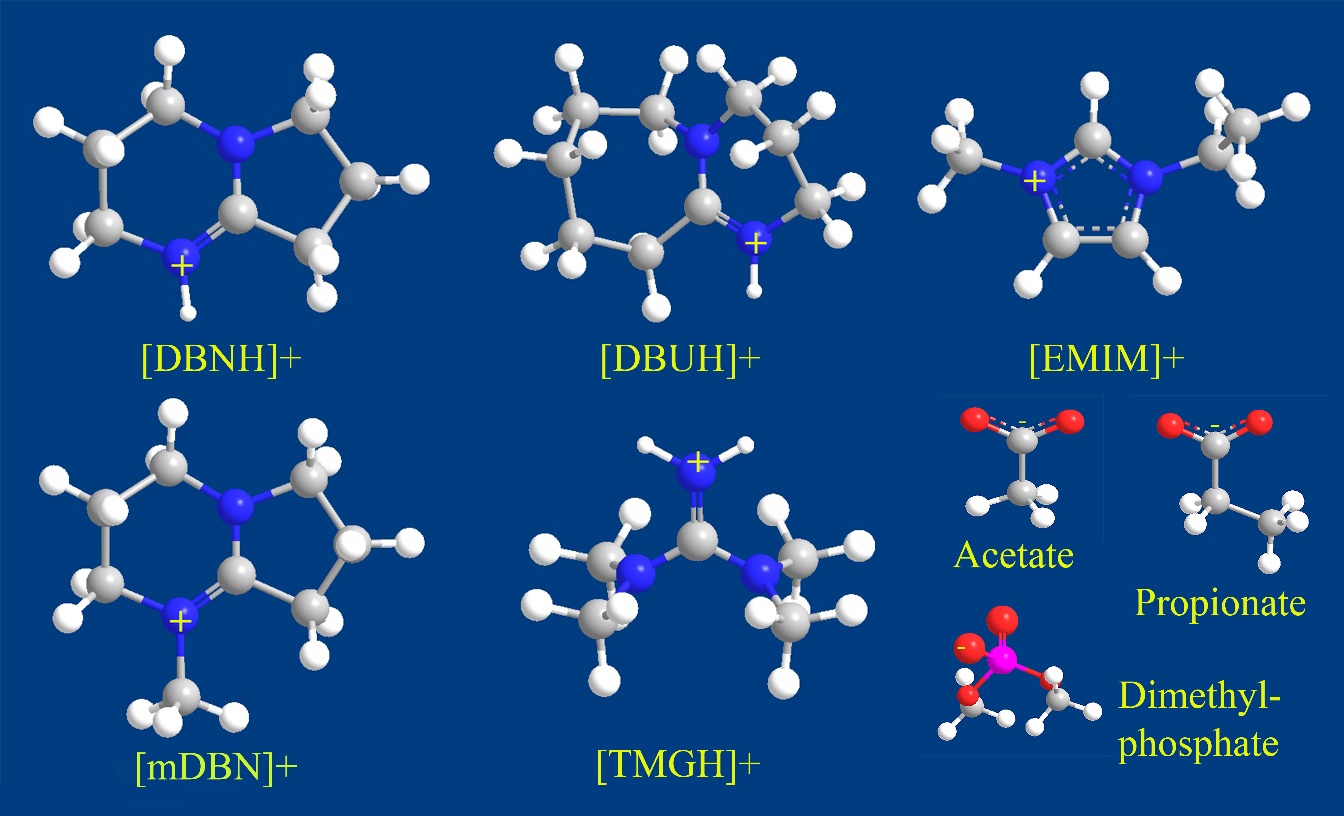

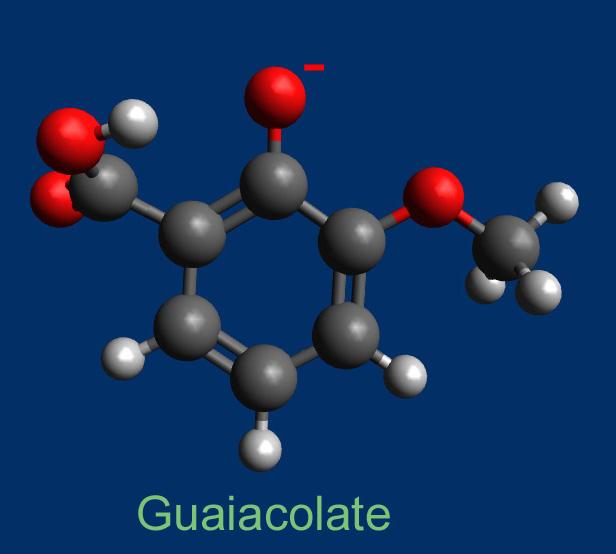


Figure 8. Structures of cations and anions used in the study. The molecules were prepared by structure minimization as reported earlier (Hebal et al., 2020).

**
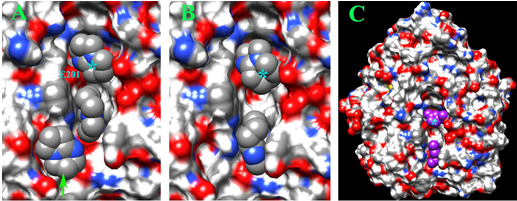
**

**Figure 9.** The active site occupation by space-filling modes of selected IL cations. The highest energy binder was placed first into the active site and then it was assessed how many other IL cations have enough space to fit in the active site. It should also be noted that it might be possible to find additional single binding sites e.g. by allowing side chain movement in the docking. A) PhEG with [DBNH]^+^, B) PhEG with [DBUH]^+^, C) PhEG with [EMIM]^+^. Stars show the highest energy binders (binding energies in Table 1). Green arrow in A shows the [DBNH]^+^ cation that covers only slightly the active site canyon. The place for the catalytic residues is shown by one catalytic residue (E201). The molecular graphics were created by UCSF Chimera (<https://www.cgl.ucsf.edu/chimera/>).


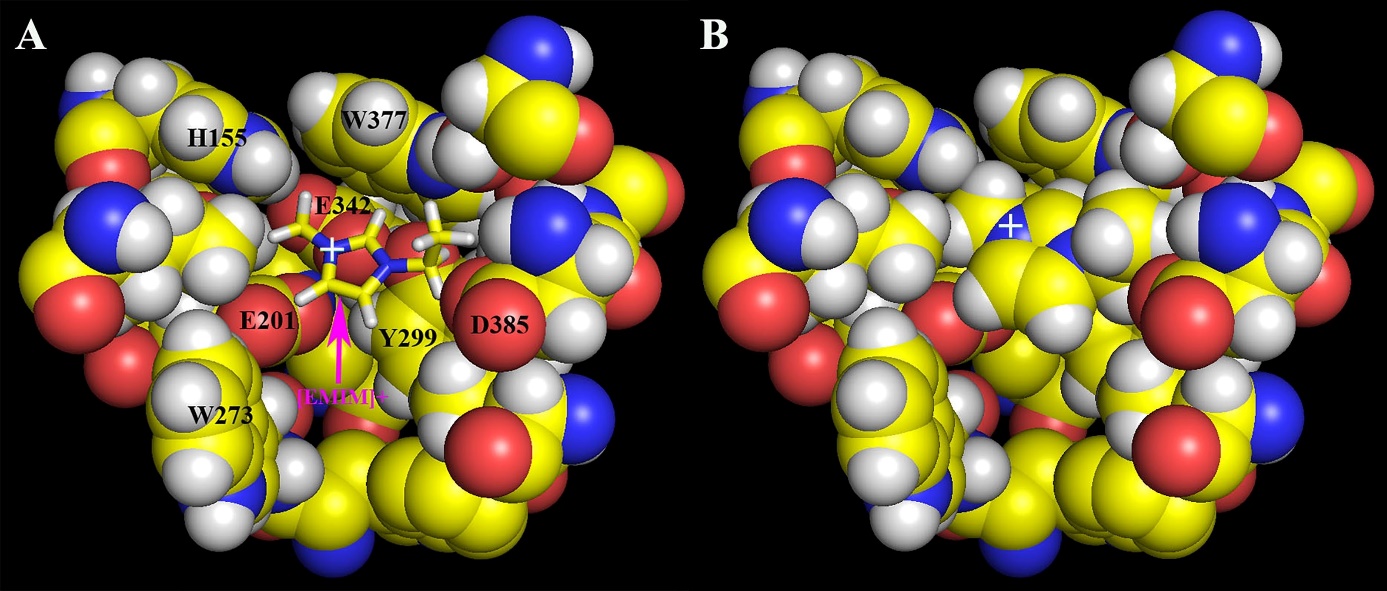


**Figure 10.** Molecular docking of [EMIM]^+^ cation into the active site of PhEG. A position for the highest energy binder of the [EMIM]^+^ cation is shown located close to the catalytic residues; estimated binding energy ∆G was -8.25 kcal/mol. A and B show space-filling structures for the active site amino acid residues: A shows [EMIM]^+^ cation in a stick mode, and B shows space-filling structure for the [EMIM]^+^ cation. E201 (acid/base) and E342 (nucleophile) are the catalytic residues. Colours of atoms: carbon, yellow; nitrogen, blue; oxygen, red, hydrogen, white. The molecular graphics were created by PyMol (<https://pymol.org/2/>).

**
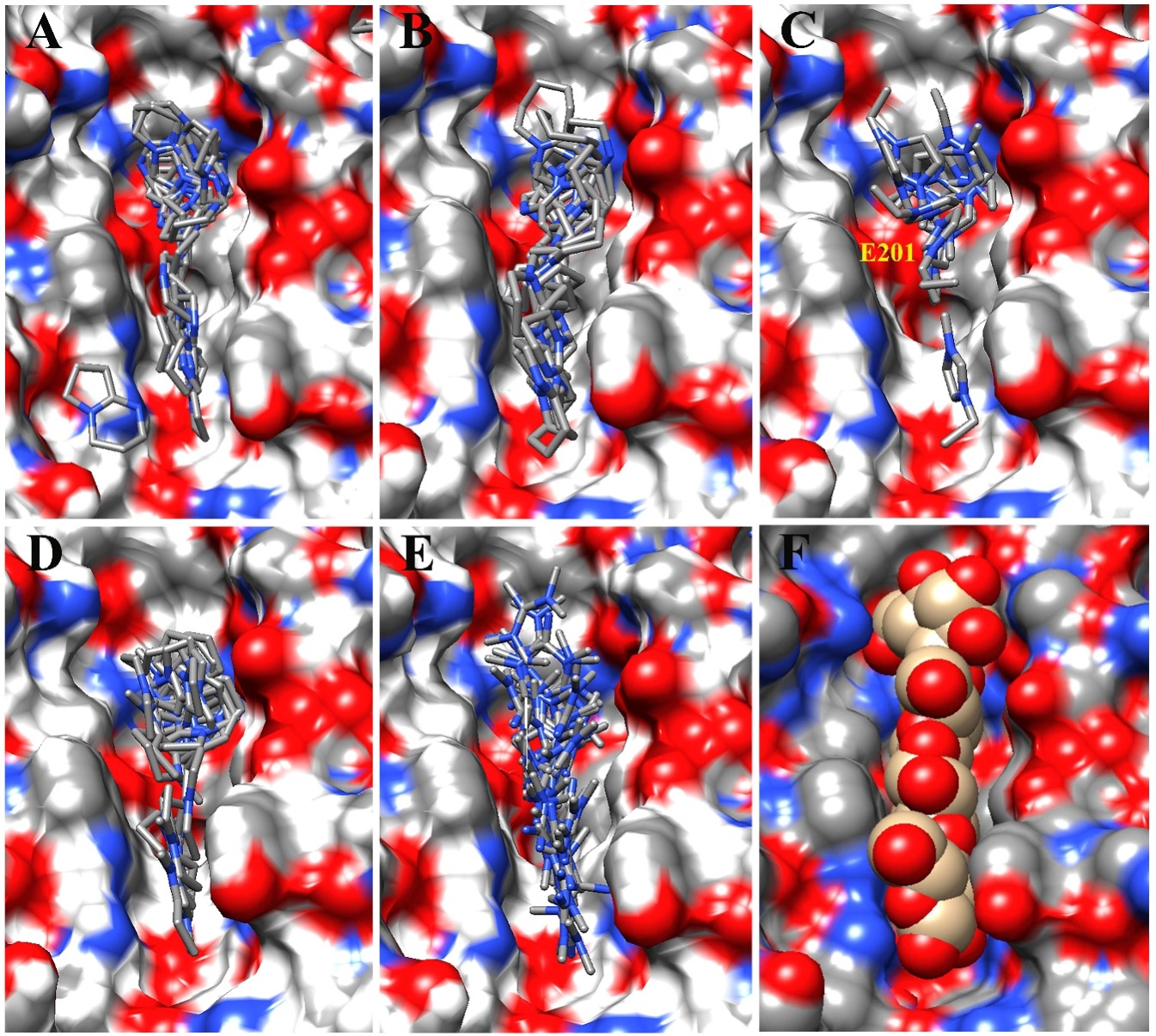
**

**Figure 11.** The potential IL cation binding sites found by docking in the active site of PhEG. The amount of overlapping binding positions probably reflects the surface area binding the IL cations. The first poses of each cluster of binding IL cations are shown in the images A-E: A) [DBNH]^+^, B) [DBUH]^+^, C) [EMIM]^+^, D) [mDBN]^+^ and E) [TMGH]^+^. F) Cellotetraose is shown in space-filling mode in the active site of PhEG. The cellotetraose structure was transferred from 3QHM to 2ZUM in SwissPdb-Viewer. The active site residues are in the middle area of cellotetraose; acid/base E201 is shown in the image C. The smaller potential binding surface for [EMIM]^+^ when compared to other IL cations is seen in C. The molecular graphics were created by UCSF Chimera (<https://www.cgl.ucsf.edu/chimera/>).


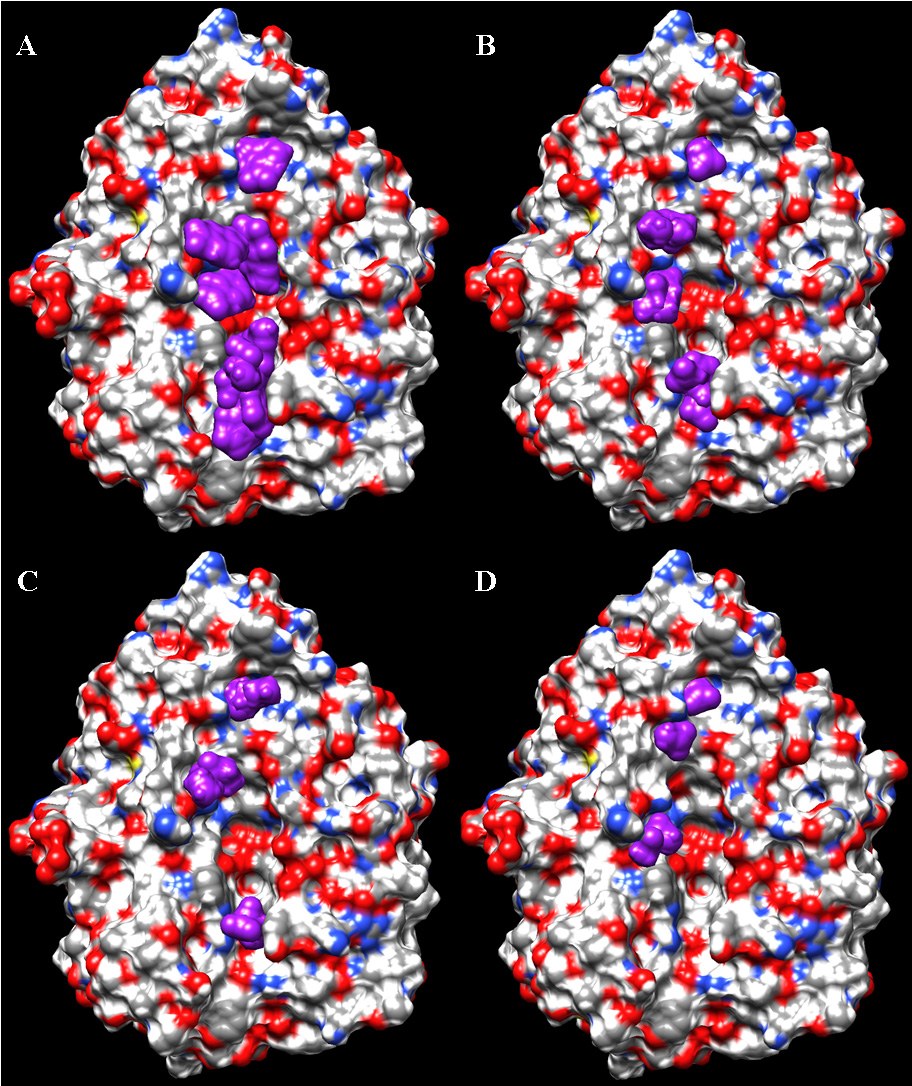


**Figure 12.** Docking of guaiacolate (A), butyrate (B), propionate (C) and acetate (D) into the active site PhEG by SwissDock. The first hit in each binding cluster is shown. The highest energy bindings were calculated for the positions in the upper edge of the active site canyon. The highest binding energies among the active site clusters (the amount of active site clusters out of all clusters is shown in parenthesis): guaiacolate 7.1 kcal/mol (12/40); butyrate 6.4 kcal/mol (9/37); propionate 6.7 kcal/mol (7/35); acetate 6.4 kcal/mol (4/35). The dockings showed that larger anion molecules are likely to fill better the active site and with higher binding energies than smaller anions. The anions produced much less active site clusters than anions (compare to Table I). Butyrate docking is shown as comparison, though no experiments were done with butyrate anions. The molecular graphics were created by UCSF Chimera (<https://www.cgl.ucsf.edu/chimera/>).

**Table 1.** Molecular docking results and PhEG activities in ILs. The amounts of clusters (each cluster contained one or more poses) binding to the active site and the whole protein in SwissDock are shown in the table. Activities are with carboxymethyl cellulose (Na-CMC).

| **IL cation** | **Active site cations^●^**  **/ ΔG***  kcal/mol | **Active site clusters**  (in bold/out of all clusters) | **STD** | ***K*_m_ with 5% IL** | ***K*_m_ with 5% IL** | ***K*_m_ with 5% IL** | **15% IL activity (%)** | | **25% IL activity (%)** | | **5% IL activity (%)** |
| --- | --- | --- | --- | --- | --- | --- | --- | --- | --- | --- | --- |
|  |  |  |  | OAc | CO_2_Et | Guaiacolate | OAc | CO_2_Et | OAc | CO_2_Et | Guaiacolate |
| **No IL** |  |  |  | 1.0 |  |  |  |  |  |  |  |
| [DBNH] | ~2 / -8.40 | 25/47=0.53 | 0.95 | 3.27 | 5.58 | 4.23 | 103.9 | 102.1 | 67.2 | 23.8 | 102,8 |
| [DBUH] | 2 / -8.38 | 19/40=0.48 | 0.91 | 13.46 | 7.7 | 8.47 | 66.9 | 76.3 | 34.2 | 23.2 | 144;8 |
| [EMIM] | 2 / -8.26 | 12/35=0.34 | 0.69 | 1.66 |  |  | 123.2 |  | 124.0 |  |  |
| [mDBN] | 2 / -8.06 | 20/47=0.43 | 0.76 |  |  |  | 117.0 |  | 39.0 |  |  |
| [TMGH] | 2 / -8.26 | 40/67=0.60^§^ | 0.96 | 4.57 | 3.76 | 11.92 | 91.0 | 92.3 | 65.4 | 85.6 | 126,9 |

**^●^** The highest energy binder was placed into the active site and then looked how many other IL cations can bind into the active site at the same time from the docked active site binders; the obtained total numbers are shown.

* Binding energy of the strongest binder. ^§^ This docking produced numerous single pose clusters. **^#^**Average ΔG is for poses shown by SwissDock (see the main text for details).
